# School-based preventive chemotherapy program for schistosomiasis and soil-transmitted helminth control in Angola: 6-year impact assessment

**DOI:** 10.1371/journal.pntd.0010849

**Published:** 2023-05-17

**Authors:** Adam W. Bartlett, Elsa P. Mendes, Latifeh Dahmash, Marta S. Palmeirim, Maria C. de Almeida, Luis B. Peliganga, Luis M. M. Lufunda, Ana Direito, Julio Ramirez, Pauline N. Mwinzi, Sergio Lopes, Susana Vaz Nery

**Affiliations:** 1 Kirby Institute, University of New South Wales, Sydney, Australia; 2 National Directorate of Public Health, Ministry of Health, Luanda, Angola; 3 The MENTOR Initiative, Huambo, Angola; 4 Swiss Tropical and Public Health Institute, Basel, Switzerland; 5 University of Basel, Basel, Switzerland; 6 Expanded Special Project for Elimination of Neglected Tropical Diseases, Brazzaville, Congo; Federal University of Agriculture Abeokuta, NIGERIA

## Abstract

**Background:**

A school preventive chemotherapy (PC) program for soil-transmitted helminths (STHs) and schistosomiasis has operated in Huambo, Uige and Zaire provinces, Angola, since 2013 and 2014, respectively; complemented by a school water, sanitation and hygiene (WASH) program in a subset of schools from 2016. Conducted in 2021, this is the first impact assessment of the school program for the control of schistosomiasis and STHs.

**Methodology/Principal findings:**

A two-stage cluster design was used to select schools and schoolchildren for parasitological and WASH surveys. The rapid diagnostic tests (RDTs), point of care circulating cathodic antigen (POC-CCA) and Hemastix, were used to estimate *Schistosoma mansoni* and *Schistosoma haematobium* prevalence, respectively. Kato Katz was used to detect STHs, and quantify STH and *S*. *mansoni* infections. Urine filtration was used to quantify *S*. *haematobium* infections. Prevalence, infection intensity, relative prevalence reduction and egg reduction rates were calculated for schistosomiasis and STHs. Cohen’s Kappa co-efficient was used to assess agreement between RDTs and microscopy. Chi-square or Fisher’s exact test was used to compare WASH indicators in WASH-supported and WASH-unsupported schools.

Overall, 17,880 schoolchildren (599 schools) and 6,461 schoolchildren (214 schools) participated in the schistosomiasis and STH surveys, respectively. Prevalence of any schistosomiasis in Huambo was 29.6%, Uige 35.4%, and Zaire 28.2%. Relative reduction in schistosomiasis prevalence from 2014 for Huambo was 18.8% (95% confidence interval (CI) 8.6, 29.0), Uige -92.3% (95%CI -162.2, -58.3), and Zaire -14.0% (95%CI -48.6, 20.6). Prevalence of any STH in Huambo was 16.3%, Uige 65.1%, and Zaire 28.2%. Relative reduction in STH prevalence for Huambo was -28.4% (95%CI -92.1, 35.2), Uige -10.7% (95%CI -30.2, 8.8), and Zaire -20.9% (95%CI -79.5, 37.8). A higher proportion of WASH-supported schools had improved water sources, and toilet and handwashing facilities compared to WASH-unsupported schools.

**Conclusions/Significance:**

The limited impact this school program has had in controlling schistosomiasis and STHs identifies the need for a comprehensive understanding of individual, community, and environmental factors associated with transmission, and consideration for a community-wide control program.

## Author summary

Schistosomiasis and soil-transmitted helminths (STHs) have long been recognized as public health problems in Angola, including Huambo, Uige and Zaire provinces. To address these neglected tropical diseases, the Angolan Ministry of Health, in conjunction with the MENTOR Initiative and END Fund have been implementing school preventive chemotherapy (PC) from 2013 for STHs and 2014 for schistosomiasis. Additionally, the school PC program has been complemented by a school WASH program in a subset of schools from 2016. To assess the impact of the school program on the prevalence and burden of schistosomiasis and STHs, parasitological surveys were conducted in 2021 that included 17,880 schoolchildren from 599 schools in the schistosomiasis survey and 6,461 schoolchildren from 214 schools in the STH survey. Access and use of WASH interventions was also assessed via questionnaires for each school participating in the parasitological surveys, and compared between WASH supported and unsupported schools. When compared to the results of the 2014 survey there has been limited reduction in the prevalence of schistosomiasis and STHs across the three provinces. A higher proportion of schools that received WASH support provided schoolchildren with better WASH conditions.

## Introduction

Schistosomiasis and soil-transmitted helminth (STH) infections are caused by parasitic worms endemic in many low-income tropical and subtropical countries, and are major contributors to the disease burden associated with neglected tropical diseases (NTDs) [1]. These NTDs have been targeted by the World Health Organization (WHO) for elimination as a public health problem by 2030, defined as a prevalence of less than 1% for heavy intensity infections for schistosomiasis, and less than 2% for moderate and heavy intensity infections for STHs [2]. The main schistosome species in Africa are *Schistosoma mansoni* and *Schistosoma haematobium*, and the main STH species globally include *Ascaris lumbricoides*, *Trichuris trichiura* and hookworms (*Necator americanus*, *Ancylostoma duodenale*, *Ancylostoma ceylanicum*). School-aged children bare a disproportionate burden of disease associated with these infections through frequent exposure to parasite eggs and larvae via contaminated soil and water and the longer-term impact on their growth and development [3]. A key strategy toward achieving WHO targets is preventive chemotherapy (PC), whereby entire or at-risk populations receive regular anthelminthic medications. The frequency of PC rounds is informed by prevalence of infection estimated through parasitological surveys [3]. While PC programs are very effective in decreasing burden of infection in the short term, for the sustainable and long-lasting control of schistosomiasis and STHs, interventions to improve access to clean water, and improve sanitation and adequate hygiene (WASH) conditions are recommended; being particularly important after PC programs are discontinued to prevent rebound of infections to pre-PC levels [4,5].

In recognition of the public health problems posed by schistosomiasis and STHs in Angola [6,7], a school PC program for STH control was initiated in the provinces of Huambo, Uige and Zaire in 2013, which was integrated with PC for schistosomiasis control from 2014. This program was subsequently informed by a prevalence survey conducted in 2014, which found a prevalence of any STH infection in Huambo of 13.1% (municipality range 0.8–33.2%), Uige 49.4% (range 5.2–89.7%), and Zaire 20.6% (range 6.7–36.8%) [8]. The prevalence of any schistosomiasis in Huambo was 34.7% (municipality range 26.9–57.0%), Uige 25.3% (range 5.9–77.3%), and Zaire 32.2% (range 6.9–51.2%) [8]. The frequency of school delivery of PC for STHs and schistosomiasis was determined at the municipality level based on these prevalence estimates in keeping with WHO recommendations [3]. Schistosomiasis prevalence was determined by rapid diagnostic tests (RDTs)–the point of care circulating cathodic antigen (POC-CCA) to detect *S*. *mansoni* and Hemastix to detect haematuria as a proxy for infection with *S*. *haematobium* (considering trace readings as positive). STH prevalence was assessed using Kato Katz. To complement the school PC program, WASH interventions were implemented in a subset of schools in Huambo, Uige and Zaire provinces from 2016. These interventions included the provision of materials for building latrines and handwashing stations, handwashing education and establishing hygiene clubs.

Impact assessments are designed to investigate the impact of control strategies, track progress toward WHO disease-specific targets, and inform subsequent control strategies [9,10]. To evaluate the impact of the school PC program for schistosomiasis and STHs across Huambo, Uige and Zaire after 6 years of operation, an impact assessment was performed throughout these provinces in 2021, with the following objectives: (i) to determine the prevalence and intensity of schistosomiasis and STH infections; (ii) compare the prevalence and intensity of schistosomiasis and STH infections with the results from the 2014 survey; and (iii) investigate student access to WASH interventions at school.

## Methods

### Ethics statement

Approval for the survey protocol was obtained by the Ministry of Public Health of Angola (17/C.E./2021) and the University of New South Wales, Sydney, Australia (HC210192). Informed written consent was obtained from the school directors of each school to allow field teams to visit. Parents/guardians at the school were then provided all the relevant study information and informed written consent was obtained by parents/guardians of schoolchildren present on the day of field teams visiting to participate in the surveys.

### Study area and setting

**Fig 1:**
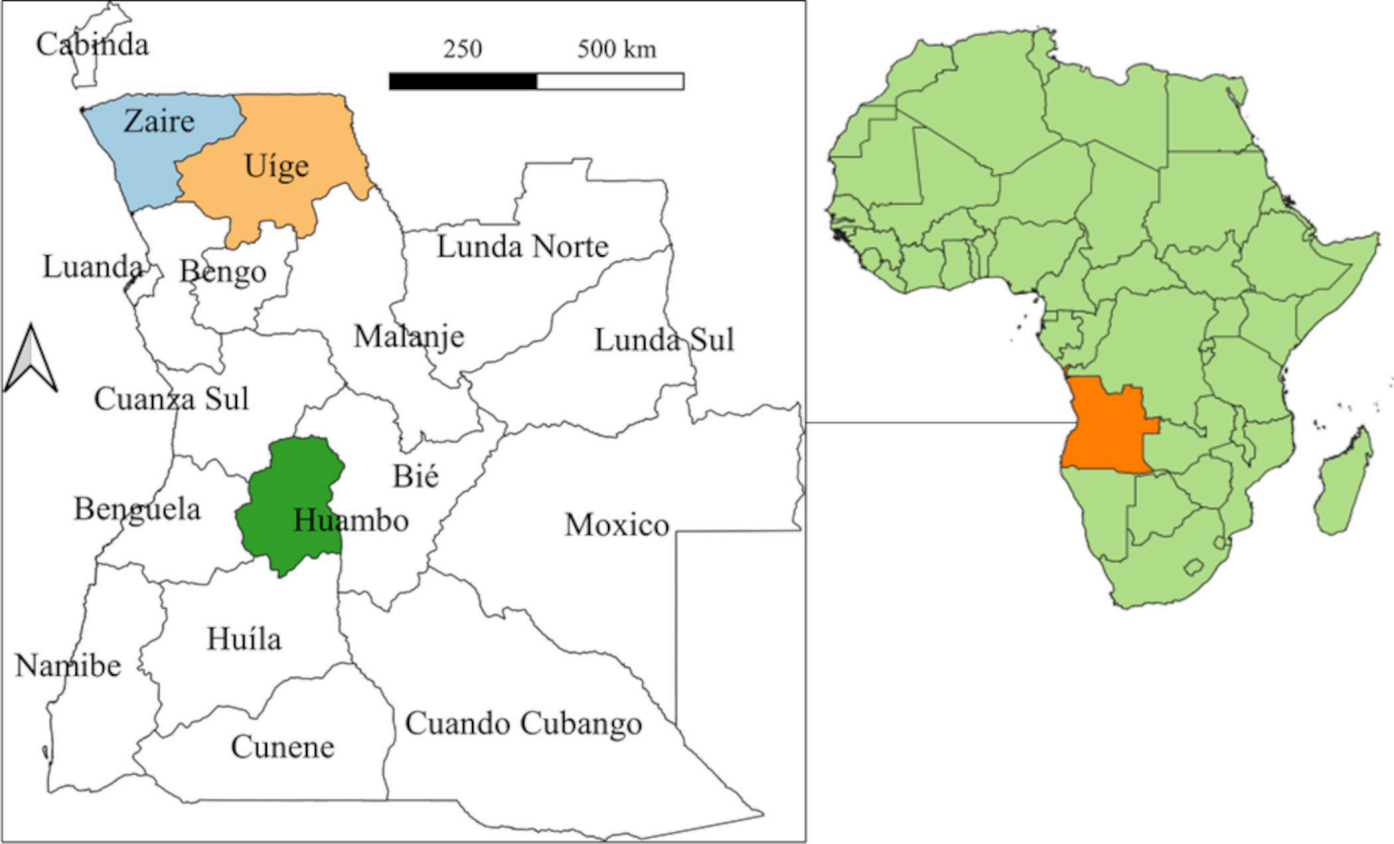
Location of Huambo, Uige and Zaire provinces, Angola. Base-layer map provided by the Database of Global Administrative Areas (GADM): https://gadm.org/download_country.html; license: https://gadm.org/license.html.

Angola is located on the west coast of southern Africa with an area of 1,246,700km^2^ [11]. It is composed of 18 provinces, 164 municipalities and 559 communes, with 91.5% of locations considered rural and 8.5% considered urban [11]. Fig 1 displays the location of Huambo, Uige and Zaire provinces in Angola. Huambo has open forests and savannah, with an annual rainfall of 1400mm and an average temperature of 18–20°C [11]. The central and western parts of Uige have similar conditions to Huambo, while eastern areas consist of dense and humid forests, with an annual rainfall of 1200-1500mm and an average temperature of 23–24°C [11]. The western parts of Zaire are similar to eastern Uige, while eastern parts of Zaire primarily consist of dense bush and savannah with an annual rainfall of <800mm and average temperature of 25–26°C [11]. There are 11 municipalities in Huambo, 16 municipalities in Uige and 6 municipalities in Zaire.

### Study design and sample size calculations

A two-stage cluster survey design was used to select schools and schoolchildren to participate in the schistosomiasis and STH parasitological surveys. Separate sample size calculations were performed for each of the parasitological surveys. For the schistosomiasis survey, sample size calculations followed a recent study that found surveying 15–20 schools per district and 20–30 children per school maximized cost efficiencies whilst minimized risk of under-treatment [12]. As such, the schistosomiasis survey aimed to obtain samples from 30 schoolchildren from 20 schools in each municipality in Huambo, 17 schools in each municipality in Uige, and 19 schools in each municipality in Zaire. To account for a 25% non-consent or urine specimen non-return rate, 38 schoolchildren from each of the selected schools were invited to provide samples.

The sample size for the STH survey was calculated using the formula: n = [DEFF*Np(1–p)]/[d^*2*^*/Z*^*2*^_1-α/2_*(N-1)+p*(1-p)] [13]; with a design effect (*DEFF*) of 2.5; total school-aged population (*N*) for Huambo of 563,301, Uige 413,790 and Zaire 165,845 (enumerated in the 2014 census); confidence interval (CI) of 95%; and an estimated prevalence (*p*) of 7% and precision (*d*) of 1.5% in Huambo, estimated prevalence (*p*) of 30% and precision (*d*) of 4% in Uige, and estimated prevalence (*p*) of 11% and precision (*d*) of 2% in Zaire. The estimated prevalence for each province was based on the 2014 prevalence of *A*. *lumbricoides* (the most prevalent STH species: 11.5% in Huambo, 49.2% in Uige, and 17.6% in Zaire) [8] with an expected prevalence reduction of 38% [14]. Cluster sizes were designated to obtain samples from a minimum of 30 schoolchildren per school, in keeping with the schistosomiasis survey. To account for a 35% non-consent or stool specimen non-return rate, 41 schoolchildren from each of the selected schools were invited to provide stool samples for the STH survey. Table 1 summarizes the sample size calculations for the schistosomiasis and STH survey.

**Table 1:** Sample size calculations for the components of the impact assessment.

	Huambo	Uige	Zaire	Total
Overall no. schools	1,023	1,278	288	2,589
**Schistosomiasis survey**				
No. schools	220	270	112	602
Planned sample size	6,600	8,100	3,360	18,060
No. students invited^a^	8,360	10,260	4,256	22,876
**STH survey**				
No. schools	95	48	81	224
Planned sample size	2,850	1,440	2,430	6,720
No. students invited^b^	3,895	1,968	3,321	9,184
**School WASH survey**				
No. schools	220	270	112	602

^a^To account for a 25% non-consent or urine specimen non-return rate. ^b^To account for a 35% non-consent or stool specimen non-return rate. RDT = rapid diagnostic test. STH = soil-transmitted helminth. WASH = water, sanitation, and hygiene.

Given the larger sample size for the schistosomiasis survey, schools were initially selected for the schistosomiasis survey via systematic random sampling from a list of all primary and combined schools in each province obtained from the Ministry of Education. To ensure representation from each municipality, sampling was stratified by municipality. For the STH survey, schools were selected via systematic random sampling from the list of schools selected for the schistosomiasis survey (stratified by municipality). For selected schools that were deemed unable to be accessed following operational assessment, substitution schools were selected via systematic random sampling from a list of remaining schools within the municipality. All sampling procedures were performed using SAS version 9.4 (SAS, Cary, NC) and involved generation of a random seed number to subsequently select schools within each municipality.

### Field operations

Field teams underwent a five-day training course covering all aspects of field work in May 2021 conducted by MSP and members from the MENTOR Initiative and Public Health National Directorate of Angola. Training material was prepared by AWB, MSP and SVN. RDT and microscopy technicians were trained by an experienced parasitologist (MSP). School directors were notified in advance of the planned arrival of field teams, who in turn notified schoolchildren and their parents/guardians to be present on the day of data collection. On the day of data collection, field teams used systematic random sampling to select either 38 schoolchildren to provide a urine specimen (for schools participating in the schistosomiasis survey only) or 41 schoolchildren to provide both a urine and stool specimen (for schools participating in both schistosomiasis and STH surveys). RDT and microscopy technicians analysed the urine and stool specimens the same day as specimen collection, and an interviewer conducted the school WASH questionnaire. Provincial supervisors provided daily supervision of all aspects of the field work to ensure operating procedures and protocols were being followed correctly.

### Diagnosis of schistosomiasis and STH infections

The diagnosis of *S*. *mansoni* and *S*. *haematobium* was assessed using urine RDTs, POC-CCA and Hemastix (to detect haematuria as a proxy for infection with *S*. *haematobium*), respectively. The POC-CCA results were classified as “negative”, “trace”, “+”, “++” and “+++” [15]. The Hemastix results were classified as “negative”, “trace not haemolysed”, “trace haemolysed”, “+”, “++” and “+++” according to manufacturer’s instructions. In schools selected for both the schistosomiasis and the STH survey, urine samples were also analysed by urine filtration microscopy to detect and quantify the number *S*. *haematobium* eggs per 10mL of urine [3]. In these schools, stool samples were analysed using the duplicate Kato Katz thick smear microscopy technique and read within 60 minutes of preparation [3] to detect and enumerate eggs for STH species (*A*. *lumbricoides*, hookworms, and *T*. *trichiura*) and *S*. *mansoni*.

### School water, sanitation and hygiene survey

The school WASH survey included questions on student access to water, toileting and handwashing facilities at school, as well as WHO/UNICEF Joint Monitoring Programme core indicators for monitoring WASH in schools [16] (S1 Information). Definitions for improved basic services for water, sanitation and hygiene were in keeping with WHO/UNICEF Joint Monitoring Programme recommendations [16].

### Data management and statistical analysis

Recruited schoolchildren were assigned a unique participant identification number (ID) and entered into registers, incorporating participant’s ID, name, consent, age, sex, and the surveys they participated in. Data for the parasitological surveys and school WASH questionnaire was entered directly into the ESPEN Collect mobile data collection tool using tablets. If tablets were unavailable or if the schools had not been pre-entered into the database (due to school substitution following operational assessment), the data was recorded in back-up paper-based forms and later entered into ESPEN Collect or transferred directly to the data management centre (Kirby Institute, UNSW Sydney, Australia). Back-up paper-based forms were required for 2,001/17,880 (11.2%) participants. Data recorded in ESPEN Collect was made available through a secure cloud-based data repository (https://metabase.espen.securedatakit.com), where access is provided to selected members of the project team.

Descriptive statistics (absolute numbers, median, interquartile range (IQR)) were used to report the characteristics of the schistosomiasis and STH parasitological survey populations. The prevalence of *Schistosoma* spp. and STHs (with 95% CIs) was calculated accounting for clustering at the school level. In addition to prevalence calculations made for individual *Schistosoma* and STH species, the prevalence of any schistosomiasis or any STH infection was calculated by combining the detection of any *Schistosoma* or any STH species using the same diagnostic tool. Graphical representation of schistosomiasis and STH prevalence was performed using QGIS version 3.18 (QGIS.org, 2022. QGIS Geographic Information System. QGIS Association. http://www.qgis.org).

For schistosomiasis, the primary prevalence estimates and relative prevalence reductions were derived from the results of the RDTs, considering trace readings as positive in keeping with manufacturer’s instructions, the Schistosomiasis Consortium for Operational Research and Evaluation recommendations (for POC-CCA) [17], and the baseline survey [8]. Prevalence estimates were also reported separately for when RDT trace readings were considered negative. The relative prevalence reduction was calculated using the formula: (*p*1−*p*2)/*p*1; where *p*1 represents the prevalence from the 2014 survey and *p*2 represents the prevalence from the 2021 survey. Generalised linear models, using the binomial family and adjusted for clustering at the school level, were used to calculate the relative prevalence reduction (with 95%CIs) between the 2021 and 2014 schistosomiasis (considering RDT trace readings as positive) and STH prevalence surveys. A positive result indicates a relative reduction in prevalence, while a negative result indicates a relative increase in prevalence. For 95%CIs with a lower bound below zero and an upper bound above zero, the result was not considered statistically significant. An appropriate response to therapy for schistosomiasis was defined as at least one third relative reduction in prevalence according to WHO recommendations [9]. A diagnostic comparison between schistosomiasis RDTs and microscopy (analysed separately when considering RDT trace readings as positive and negative) was performed using Cohen’s Kappa co-efficient (very good, κ>0.8; good, 0.6<κ≤0.8; moderate, 0.4<κ≤ 0.6; fair, 0.2<κ≤0.4; poor, κ≤0.2).

Classification of infection intensity (low, moderate or high) for STH and *Schistosoma* species followed WHO criteria [3]. The egg reduction rate (ERR) was measured using the formula: [mean infection intensity at baseline (2014 survey)–mean infection intensity at follow-up (2021 survey)] / mean intensity at baseline (2014 survey) [18]; with 95%CIs estimated using bootstrapping with 10,000 repetitions and adjusted for clustering at the school level. A positive result indicates a reduction in egg count, whilst a negative result indicates an increase in egg count. For 95%CIs with a lower bound below zero and an upper bound above zero, the result was not considered statistically significant.

For the school WASH survey, descriptive statistics were used to describe the number and proportion of schools with evidence related to WASH indicators for schools supported by the WASH program (WASH-supported) and schools that were not (WASH-unsupported) across Huambo, Uige and Zaire. A chi-square or Fisher’s exact test was performed to assess for differences in WASH indicators for WASH-supported and WASH-unsupported schools. All statistical analyses were performed using STATA version 17.0 (College Station, Texas).

## Results

### Survey population

Overall, 17,880 schoolchildren from 599 schools participated in the schistosomiasis survey, and 6,461 schoolchildren from 214 schools participated in the STH survey (Table 2). For those whom demographic data was available the median age was 9 years (IQR 7, 11) for both the schistosomiasis and STH surveys (data available for 16,384 and 6,226 schoolchildren respectively). There was comparable participation of males and females for both the schistosomiasis (males 9,183/16,910, 54.3%) and STH (males 3,294/6,246, 52.7%) surveys.

**Table 2:** Demographic characteristics of the schistosomiasis and soil-transmitted helminth survey.

	Schistosomiasis survey				STH survey			
	**Schools**	**Students**	**Sex**^a^ **(M/F)**	**Age**^b^ **Median (IQR)**	**Schools**	**Students**	**Sex**^a^ **(M/F)**	**Age**^b^ **Median (IQR)**
Huambo	221	6,591	3,355/3,171	9 (7, 11)	100	2,998	1,452/1,422	9 (7, 11)
Uige	266	7,963	4,363/3,286	9 (7, 11)	46	1,419	767/646	9 (7, 11)
Zaire	112	3,326	1,465/1,270	9 (7, 11)	68	2,044	1,075/913	9 (7, 11)
**Total**	**599**	**17,880**	**9,183/7,727**	**9 (7, 11)**	**214**	**6,461**	**3,294/2,981**	**9 (7, 11)**

^a^Demographic data not available for all participants. ^b^Data available for 16,384 and 6,226 participants in the schistosomiasis and STH surveys, respectively. F = female. IQR = interquartile range. M = male. STH = soil-transmitted helminth.

### Schistosomiasis survey

**Fig 2:**
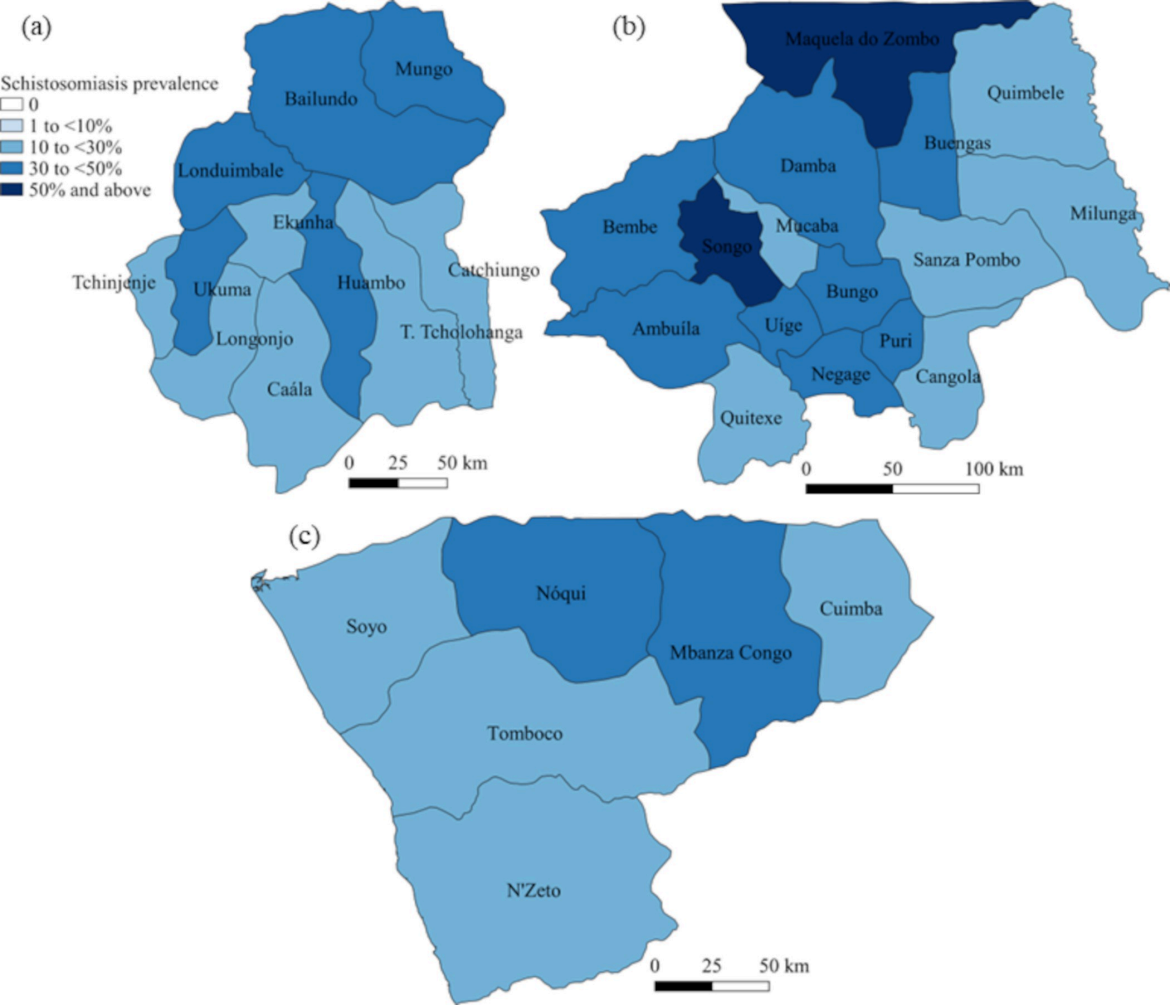
Prevalence of schistosomiasis for each municipality in (a) Huambo, (b) Uige and (c) Zaire provinces, Angola. Base-layer map provided by the Database of Global Administrative Areas (GADM): https://gadm.org/download_country.html; license: https://gadm.org/license.html.

The provincial prevalence of schistosomiasis as determined by RDTs, when considering trace readings as positive, are reported in Table 3 and presented in Fig 2. The overall prevalence of schistosomiasis for Huambo was 29.6% (municipality range 20.7–39.7%), Uige 35.4% (range 17.0–53.9%), and Zaire 28.2% (range 17.9–41.2%). When compared to the baseline survey, the relative prevalence reduction in schistosomiasis for Huambo was 18.8% (95%CI 8.6, 29.0), Uige -92.3% (95%CI -126.2, -58.3), and Zaire -14.0% (95%CI -48.6, 20.6) (Table 3). Results for each municipality are reported in S2 Information. There were only four municipalities in Huambo that achieved an appropriate response (at least one third reduction in relative prevalence) [9], with no municipalities in Uige or Zaire demonstrating an appropriate response.

**Table 3:** Prevalence of schistosomiasis using rapid diagnostic tests for each province and comparisons with the baseline survey.

	Impact assessment				Prevalence comparison for any schistosomiasis	
	**Schools / Students**	***Schistosoma mansoni***	***Schistosoma haematobium***	**Any schistosomiasis**	**Any schistosomiasis at baseline**	**Relative prevalence reduction**
	**N/N**	**% (95%CI)**	**% (95%CI)**	**% (95%CI)**	**% (95%CI)**	**% (95%CI)**
Huambo	221/6,591	23.1 (20.8, 25.5)	9.4 (7.9, 11.1)	29.6 (27.1, 32.2)	36.4 (33.1, 39.8)	**18.8 (8.6, 29.0)**
Uige	266/7,963	29.6 (26.6, 32.8)	7.8 (6.5, 9.4)	35.4 (32.5, 38.5)	18.4 (15.8, 21.4)	**-92.3 (-126.2, -58.3)**
Zaire	112/3,326	19.7 (16.5, 23.3)	13.2 (9.2, 18.4)	28.2 (23.6, 33.4)	24.8 (18.9, 31.7)	-14.0 (-48.6, 20.6)

Prevalence calculations based on rapid diagnostic tests (considering trace readings as positive) and adjusted for clustering at school level. Relative prevalence reduction = (2014 prevalence– 2021 prevalence) / 2014 prevalence; negative values represent a relative increase in prevalence and positive values represent a relative reduction in prevalence. CI = confidence interval. N = number surveyed.

Microscopy consistently detected a lower prevalence of schistosomiasis compared to RDTs, with the prevalence of schistosomiasis by microscopy for Huambo being 2.5% (95%CI 1.5, 4.1), Uige 5.4% (95%CI 2.7, 10.4) and Zaire 1.7% (95%CI 0.6, 4.4) (S3 Information). The prevalence of heavy intensity *S*. *mansoni* infections was 0.07% in Huambo, 1.4% in Uige and 0.08% in Zaire (Table 4). The prevalence of heavy intensity *S*. *haematobium* infections was 0.4% in Huambo and 0.2% in Zaire, with no heavy intensity *S*. *haematobium* infections detected in Uige (Table 4). Compared to baseline measurements, significant reductions in intensity of infection were only found for *S*. *haematobium* in Huambo (ERR 91.2%; 95%CI 80.5, 101.9) and Uige (ERR 98.3%; 95%CI 34.8, 161.8) (Table 4).

**Table 4:** Schistosomiasis infection intensity and egg reduction rates in Huambo, Uige and Zaire as determined by microscopy.

	Huambo	Uige	Zaire
***S*. *mansoni***			
*Prevalence*, *%(95%CI)*			
Light intensity	0.2 (0.05, 0.6)	2.1 (0.9, 5.0)	0.7 (0.1, 3.0)
Moderate intensity	0.1 (0.05, 0.4)	1.7 (0.6, 4.6)	0.2 (0.02, 1.2)
Heavy intensity	0.07 (0.02, 0.3)	1.4 (0.5, 4.0)	0.08 (0.02, 0.3)
*Intensity comparison*			
IA mean epg (95%CI)	342.5 (80.3, 604.8)	337.7 (225.5, 449.9)	139.1 (25.9, 252.2)
Baseline mean epg (95%CI)	36.0 (0, 188.5)	310.4 (248.4, 372.4)	144^a^
Egg reduction rate (95%CI)	-8.5 (-18.3, 1.3)	-0.1 (-0.9, 0.7)	0.03 (-2.5, 2.5)
***S*. *haematobium*, %(95%CI)**			
*Prevalence*, *%(95%CI)*			
Light intensity	1.7 (1.0, 3.1)	0.2 (0.06, 0.7)	0.5 (0.2, 1.2)
Heavy intensity	0.4 (0.1, 0.9)	0	0.2 (0.03, 0.8)
*Intensity comparison*			
IA mean epg (95%CI)	21.8 (14.8, 28.9)	9.7 (0, 34.2)	119.1 (0, 242.9)
Baseline mean epg (95%CI)	248.6 (123.1, 374.2)	563.2 (0, 1,722.8)	107.0 (61.6, 152.4)
Egg reduction rate, %(95%CI)	**91.2 (80.5, 101.9)**	**98.3 (34.8, 161.8)**	-0.1 (-66.8, 66.6)

^a^95% confidence interval could not be calculated due to only one observation contributing data. Egg reduction rate = (mean intensity in 2014 –mean intensity in 2021) / mean intensity in 2014; positive values represent a relative reduction in mean intensity and negative values represent a relative increase in mean intensity. Prevalence calculations adjusted for clustering at school level. CI = confidence interval. epg = eggs per gram. IA = impact assessment.

The diagnostic agreement between schistosomiasis RDTs and microscopy is shown in Table 5. When considering RDT trace readings as positive, the diagnostic agreement between POC-CCA and Kato Katz in detecting *S*. *mansoni* infection was poor (κ = 0.058, p<0.001), similarly the diagnostic agreement between Hemastix and urine filtration in detecting *S*. *haematobium* was poor (κ = 0.13, p<0.001). A similar diagnostic agreement between RDTs and microscopy was found when considering RDT trace readings as negative (Table 5).

**Table 5:** Diagnostic performance between schistosomiasis rapid diagnostic tests and microscopy.

	**RDTs (trace positive)**					
	**Detected**	**Not detected**	**Expected agreement**	**Observed agreement**	**Kappa statistic** ^**a**^	**p-value**
***S*. *mansoni***						
Microscopy			75.7%	77.1%	0.058	<0.001
Detected	71/1,564	33/5,098				
Not detected	1,493/1,564	5,065/5,098				
***S*. *haematobium***						
Microscopy			89.0%	90.5%	0.13	<0.001
Detected	56/665	20/5,918				
Not detected	609/665	5,898/5,918				
	**RDTs (trace negative)**					
	**Detected**	**Not detected**	**Expected agreement**	**Observed agreement**	**Kappa statistic** ^**a**^	**p-value**
***S*. *mansoni***						
Microscopy			90.6%	92.3%	0.18	<0.001
Detected	65/538	39/6,124				
Not detected	473/538	6,085/6,124				
***S*. *haematobium***						
Microscopy			94.0%	95.2%	0.19	<0.001
Detected	42/326	34/6,257				
Not detected	284/326	6,223/6,257				

^a^Kappa agreement classification: ≤0.20 = poor; 0.21–0.40 = fair; 0.41–0.60 = moderate; 0.61–0.80 = good; 0.81–1.00 = very good. RDTs = rapid diagnostic tests.

### Soil-transmitted helminth survey

**Fig 3:**
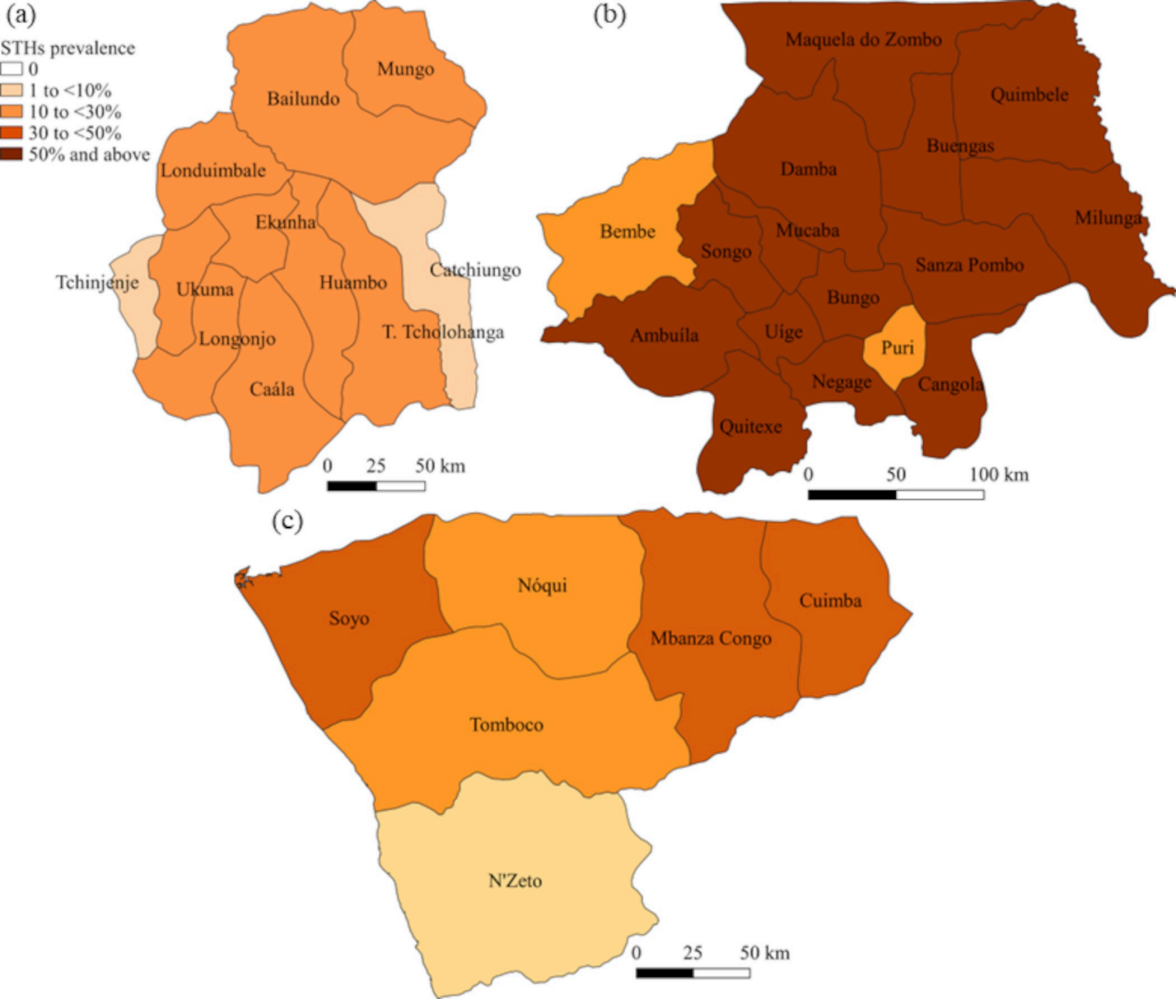
Prevalence of soil-transmitted helminths (STHs) for each municipality in (a) Huambo, (b) Uige and (c) Zaire provinces, Angola. Base-layer map provided by the Database of Global Administrative Areas (GADM): https://gadm.org/download_country.html; license: https://gadm.org/license.html.

The provincial prevalence of STH infections are reported in Table 6 and presented in Fig 3. The highest overall STH prevalence was in Uige with 65.1% (municipality range 26.7–95.6%), followed by Zaire with 28.2% (range 7.2–49.2%), then Huambo with 16.3% (range 4.1–29.2%). *Ascaris lumbricoides* was the predominant STH species across all three provinces (Table 6). When compared to the baseline survey the relative prevalence reduction in any STH infection for Huambo was -28.4% (95%CI -92.1, 35.2), Uige -10.7% (95%CI -30.2, 8.8), and Zaire -20.9% (95%CI -79.5, 37.8) (Table 6). Results for each municipality are reported in S4 Information. Only the municipality of Puri (Uige province) demonstrated a significant reduction in prevalence. Most STH infections across all three provinces were light intensity, except in Uige where *A*. *lumbricoides* moderate intensity infections were comparable to light (Table 7). A significant decrease in intensity of infection was found for *A*. *lumbricoides* and hookworm in Huambo (ERR 56.1% (95%CI 37.1, 75.0) and 71.7% (95%CI 49.6, 93.9), respectively). In other provinces there was a trend towards an increase in intensity of infection in all STH species but not statistically significant (Table 7).

**Table 6:** Prevalence of soil-transmitted helminth infections for each province and comparison with the baseline prevalence survey.

	Impact assessment					Prevalence comparison	
	Schools / students	*Ascaris lumbricoides*	Hookworm	*Trichuris trichiura*	Any STH	Any STH at baseline	Relative prevalence reduction for any STH
	N/N	% (95%CI)	% (95%CI)	% (95%CI)	% (95%CI)	% (95%CI)	% (95%CI)
Huambo	100/2,998	11.9 (9.4, 15.0)	3.4 (2.3, 5.2)	2.3 (1.7, 3.2)	16.3 (13.7, 19.4)	12.7 (7.8, 20.1)	-28.4 (-92.1, 35.2)
Uige	46/1,419	62.9 (55.0, 70.2)	3.6 (1.5, 8.6)	7.5 (4.0, 13.7)	65.1 (57.2, 72.3)	58.8 (50.5, 66.6)	-10.7 (-30.2, 8.8)
Zaire	80/2,404	23.7 (18.6, 29.6)	3.0 (1.5, 5.9)	6.4 (4.6, 8.8)	28.2 (22.8, 34.3)	23.3 (13.3, 37.6)	-20.9 (-79.5, 37.8)

Prevalence calculations adjusted for clustering at school level. Relative prevalence reduction = (2014 prevalence– 2021 prevalence) / 2014 prevalence; negative values represent a relative increase in prevalence and positive values represent a relative reduction in prevalence. CI = confidence interval. STH = soil-transmitted helminth.

**Table 7:** Soil-transmitted helminth infection intensity and egg reduction rates in Huambo, Uige and Zaire.

	Huambo	Uige	Zaire
***A*. *lumbricoides***			
*Prevalence*, *%(95%CI)*			
Light intensity	8.1 (6.4, 10.2)	38.0 (33.6, 42.5)	17.8 (14.1, 22.2)
Moderate intensity	3.8 (2.6, 5.6)	24.0 (18.5, 30.5)	5.8 (3.6, 9.2)
Heavy intensity	0.03 (0.004, 0.3)	1.1 (0.2, 4.8)	0.1 (0.01, 0.6)
*Intensity comparison*			
IA mean epg (95%CI)	5,900.2 (4,844.9, 6,955.6)	11,303.6 (6,873.2, 15,734.1)	4,426.1 (3,837.7, 5,014.4)
Baseline mean epg (95%CI)	13,430.0 (9,882.9, 16,977.2)	9,861.5 (7,867.6, 11,855.5)	1,502.1 (586.3, 2,417.8)
ERR, %(95%CI)	**56.1 (37.1, 75.0)**	-14.6 (-128.3, 99.0)	-194.7 (-847.9, 458.6)
**Hookworm**			
*Prevalence*, *%(95%CI)*			
Light intensity	3.4 (2.2, 5.1)	2.2 (1.2, 4.3)	3.0 (1.5, 5.9)
Moderate intensity	0.03 (0.004, 0.2)	0.1 (0.01, 0.5)	0.04 (0.01, 3.0)
Heavy intensity	0.03 (0.004, 0.2)	0	0
*Intensity comparison*			
IA mean epg (95%CI)	268.0 (65.4, 470.5)	311.3 (132.1, 490.4)	214.7 (122.6, 306.8)
Baseline mean epg (95%CI)	948.0 (0, 10,248.9)	212.7 (163.9, 261.5)	87.0 (53.4, 120.6)
ERR, %(95%CI)	**71.7 (49.6, 93.9)**	-46.3 (-132.8, 40.1)	-146.8 (-618.7, 325.2)
***T*. *trichiura***			
*Prevalence*, *%(95%CI)*			
Light intensity	1.5 (1.1, 2.2)	7.3 (3.9, 13.3)	6.2 (4.4, 8.5)
Moderate intensity	0.7 (0.4, 1.2)	0.4 (0.2, 1.2)	0.2 (0.1, 0.4)
Heavy intensity	0.1 (0.02, 0.4)	0	0.04 (0.01, 0.3)
*Intensity comparison*			
IA mean epg (95%CI)	4,268.3 (0, 8,882.4)	377.9 (162.0, 593.9)	203.1 (63.8, 342.4)
Baseline mean epg (95%CI)	12,342.4 (0, 24,989.5)	183.0 (41.7, 324.3)	102.0 (51.2, 152.8)
ERR, %(95%CI)	65.4 (-661.1, 791.9)	-106.5–389.2, 176.2)	-99.1 (-371.6, 173.5)

ERR = Egg reduction rate = (mean intensity in 2014 –mean intensity in 2021) / mean intensity in 2014; positive values represent a relative reduction in mean intensity and negative values represent a relative increase in mean intensity. Prevalence calculations adjusted for clustering at school level. CI = confidence interval. epg = eggs per gram. IA = impact assessment.

### School water, sanitation and hygiene survey

There were 589/599 (98.3%) schools that completed the WASH survey. Of these, there were 178 (30.2%) schools that received support from the provincial or municipality health department and operational partners to implement WASH interventions (WASH-supported) and 411 (69.8%) schools that did not receive such support (WASH-unsupported). Year-round access to water was reported by 442/589 (75.0%) schools, with similar proportions amongst WASH-supported (142/178, 79.8%) and WASH-unsupported (300/411, 73.0%) schools (Table 8). A higher proportion of WASH-supported schools reported an improved water source compared to WASH-unsupported schools (42.7% vs 28.5%, p = 0.003). A higher proportion of WASH-supported schools also had someone responsible for fetching water (78.7% vs 70.8%, p = 0.005), ability to store water (87.1% vs 77.4%, p = 0.01) and treat water (90.5% vs 78.1%, p = 0.002). A similar proportion of WASH-supported and WASH-unsupported schools were found to be free of faeces on the school grounds (83.2% vs 80.1%, p = 0.62) and free of urine (79.2% vs 75.9%, p = 0.47). There was a higher proportion of WASH-supported schools with improved toilet facilities compared to WASH-unsupported schools (69.1% vs 41.6%, p<0.001). For the hygiene indicators, there was a higher proportion of WASH-supported schools that had improved handwashing facilities (95.5% vs 81.0%, p = <0.001) and a hygiene club (66.9% vs 49.6%, p<0.001) compared to WASH-unsupported schools. S5 Information describes full results from the WASH surveys.

**Table 8:** Select results from the school water, sanitation and hygiene (WASH) questionnaire for WASH-supported and WASH-unsupported schools.

	Overall	WASH supported	WASH unsupported	P-value^a^
**Schools (N)**	589	178	411	
**Students per school, mean (range)**	593 (2–4,467)	508 (2–4,467)	554 (30–4,195)	
**Setting, %(n)**				
** Rural**	68.9 (405)	72.5 (129)	67.2 (276)	
** Urban**	31.1 (183)	27.5 (49)	32.6 (134)	
** Missing**	0.2 (1)	0	0.2 (1)	
**Water indicators**				
**Water availability, %(n)**				
All the time	75.0 (442)	79.8 (142)	73.0 (300)	0.08
During wet season only	8.0 (47)	6.7 (12)	8.5 (35)	0.47
Never	6.3 (37)	5.6 (10)	6.6 (27)	0.66
Other	3.4 (20)	3.4 (6)	3.4 (14)	0.98
Don’t know / no reply	7.3 (43)	4.5 (8)	8.5 (35)	0.09
**Type of water source, %(n)**				**0.003**
Improved	32.8 (193)	42.7 (76)	28.5 (117)	
Non-improved	64.5 (380)	55.1 (98)	68.6 (282)	
Don’t know / no reply	2.7 (16)	2.3 (4)	2.9 (12)	
**Someone responsible for fetching water, %(n)**				**0.005**
Yes	73.2 (431)	78.7 (140)	70.8 (291)	
No	20.7 (122)	19.7 (35)	21.2 (87)	
Don’t know / no reply	6.1 (36)	1.7 (3)	8.0 (33)	
**Water stored, %(n)**				**0.01**
Yes	80.3 (473)	87.1 (155)	77.4 (318)	
No	16.3 (96)	11.8 (21)	18.3 (75)	
Don’t know / no reply	3.4 (20)	1.1 (2)	4.4 (18)	
**Treat/boil water, %(n)**				**0.002**
Yes	81.8 (482)	90.5 (161)	78.1 (321)	
No	13.9 (82)	7.9 (14)	16.6 (68)	
Don’t know / no reply	4.2 (25)	1.7 (3)	5.4 (22)	
**Agent for treating water, %(n)**				
Household bleach	80.7 (475)	89.9 (160)	76.7 (315)	**<0.001**
Boil	2.7 (16)	2.8 (5)	2.9 (11)	0.93
Filter	0.2 (1)	0.6 (1)	0	0.30
Other	0.2 (1)	0	0.2 (1)	1.0
Don’t know / no reply	18.9 (111)	9.6 (17)	22.9 (94)	<0.001
**Purpose of water, % (n)**				
Drinking	43.0 (253)	57.3 (102)	36.7 (151)	**<0.001**
Handwashing	78.8 (464)	84.8 (151)	76.2 (313)	**0.02**
Cleaning	55.2 (325)	70.8 (126)	48.4 (199)	**<0.001**
Don’t know / no reply	17.8 (105)	9.6 (17)	21.4 (88)	0.001
**Sanitation indicators**				
**School free of faeces, %(n)**				0.62
Yes	81.0 (477)	83.2 (148)	80.1 (329)	
No	15.1 (89)	12.9 (23)	16.1 (66)	
No reply / not observed	3.9 (23)	3.9 (7)	3.9 (16)	
**School free of urine, %(n)**				0.47
Yes	76.9 (453)	79.2 (141)	75.9 (312)	
No	18.2 (107)	17.4 (31)	18.5 (76)	
No reply / not observed	4.9 (29)	3.4 (6)	5.6 (23)	
**Toilets available, %(n)**				**<0.001**
Yes	60.3 (355)	78.1 (139)	52.6 (216)	
No	38.0 (224)	21.4 (38)	45.3 (186)	
Don’t know / no reply	1.7 (10)	0.6 (1)	2.2 (9)	
**Toilets functional, %(n)**	N = 355	N = 139	N = 216	0.41
All	75.2 (267)	77.0 (107)	74.1 (160)	
Some	14.7 (52)	15.8 (22)	13.9 (30)	
None	3.9 (14)	3.6 (5)	4.2 (9)	
No reply / not observed	6.2 (22)	3.6 (5)	7.9 (17)	
**Type of toilet, %(n)**				**<0.001**
Improved	49.9 (294)	69.1 (123)	41.6 (171)	
Non-improved	40.8 (240)	21.9 (39)	48.9 (201)	
Don’t know / no reply	9.3 (55)	9.0 (16)	9.5 (39)	
**Hygiene indicators**				
**Handwashing facilities available, %(n)**				**0.003**
Yes	90.3 (532)	96.6 (172)	87.6 (360)	
No	8.0 (47)	2.8 (5)	10.2 (42)	
Don’t know / No reply	1.7 (10)	0.6 (1)	2.2 (9)	
**Type of handwashing facility, %(n)**				**<0.001**
Improved	85.4 (503)	95.5 (170)	81.0 (333)	
Non-improved	12.9 (76)	4.5 (8)	16.6 (68)	
Don’t know / no reply	1.7 (10)	0	2.4 (10)	
**Handwashing elements, %(n)**				0.12
Improved	50.6 (298)	55.6 (99)	48.4 (199)	
Non-improved	46.7 (275)	43.3 (77)	48.2 (198)	
Don’t know / no reply	2.7 (16)	1.1 (2)	3.4 (14)	
**Hygiene club available, %(n)**				**<0.001**
Yes	54.8 (323)	66.9 (119)	49.6 (204)	
No	43.5 (256)	32.6 (58)	48.2 (198)	
Don’t know / no reply	1.7 (10)	0.6 (1)	2.2 (9)	

More than one response was possible for some items. ^a^Chi-square or Fisher’s exact test performed to compare responses between WASH-supported and WASH-unsupported schools. WASH = water, sanitation and hygiene.

## Discussion

This is the first impact assessment of a school program for schistosomiasis and STH control in Angola. Despite periodic PC administration for STHs since 2013 and schistosomiasis since 2014, the PC program had a limited impact on the prevalence of these infections amongst school-aged children. Across all three provinces, there were only four (out of 33) municipalities that demonstrated an appropriate response to schistosomiasis control measures (as at least one third relative prevalence reduction) [9] and one municipality with a significant reduction in STH prevalence. There is also a concerningly high prevalence and burden of STH infections in Uige, where 14 municipalities had a STH prevalence ≥50%, and the provincial prevalence of moderate or high intensity infections was 25%. Impact assessments of other control programs for schistosomiasis and STHs have demonstrated a variable response [19–24], with comparisons between programs difficult to make due to differences in the frequency of drug administration and model of drug delivery (school vs community), PC coverage, inclusion of other control measures (e.g., health education, WASH interventions, and snail control), and the method of infection detection (e.g., microscopy methods or rapid diagnostic tools).

The limited impact of this school program in reducing schistosomiasis or STH infections is likely multifactorial. Firstly, whether the program coverage has been adequate needs further investigation. Coverage data, based on pills administered relative to census population data, collected by operational partners throughout the program indicate Huambo and Uige met the WHO coverage target of 75%, while Zaire fell below this target. This program also collected coverage data based on pills delivered relative to the number of enrolled schoolchildren at time of drug delivery, which mostly showed higher program coverage. Indeed, despite primary school being compulsory in Angola, the number of children not enrolled or not attending school remains uncertain, and the lack of impact of the PC program suggests the reported level of coverage may be an overestimate. Secondly, high re-infection rates may be contributing to the limited impact, with transmission occurring within the school-aged population and in the community from other age groups that have not been targeted by control measures [25,26]. Finally, there may be a lack of access to improved water, sanitation and hygiene measures at home, and the majority of schools had not received WASH support.

The provincial prevalence of moderate and high intensity STH infections are above 2% for all provinces, and particularly high in Uige, indicating that WHO STH targets for elimination as a public health problem have not been met. For schistosomiasis, the low prevalence of heavy intensity infections needs to be considered in the context of the large difference in prevalence when comparing RDTs and microscopy, raising concerns for the performance of microscopy. The poor diagnostic agreement between schistosomiasis microscopy and RDTs is likely in part due to the high proportion of results “not detected” for both methods, which results in a high expected agreement and therefore a high likelihood that any observed agreement could be due to chance. Additional surveys are required to further investigate the quality of microscopy and investigate progress towards elimination as a public health problem for both schistosomiasis and STH infections.

Besides the suspected poor sensitivity of microscopy, we cannot exclude selection bias given that only children whose parents were present to provide consent were invited to participate and suspect that measures imposed during the COVID-19 pandemic would have increased absenteeism, thereby reducing the pool of students from which participants were selected. This study did not aim to assess the potential contribution of the WASH program to changes in schistosomiasis or STH prevalence given that WASH-supported schools were the minority and studies have highlighted the challenges in assessing the impact of WASH in the presence of deworming programs [4]. However, given the school WASH survey showed a higher proportion of WASH-supported schools provided an improved water source and toileting and handwashing facilities, can store and treat water, and support a hygiene club; expansion of the school WASH program throughout these provinces should be explored.

Given out results, in addition to further investigations into the real coverage of the school PC program to reflect the actual number of children at risk, considerations should be made to either increase the frequency of school PC or expand the program to provide community-wide mass drug administration (MDA). The latest WHO schistosomiasis guidelines advocate for praziquantel MDA to all those aged 2 years or more [9]. Similar considerations should be made for expanding the STH PC program to community-wide deworming, which has been found to be more impactful in reducing STH prevalence in school-aged children compared to targeted deworming programs [14]. As such, MDA for schistosomiasis and STH control could be integrated with MDA for other NTDs present in Angola such as onchocerciasis, lymphatic filariasis or trachoma.

## Conclusions

Despite the implementation of a school program for STH and schistosomiasis control since 2013 and 2014, respectively, there has been limited impact in reducing the prevalence of these NTDs in school-aged children across Huambo, Uige and Zaire provinces. Potential factors limiting the impact of the program, including inadequate treatment coverage, re-infections within the school population or from other sub-populations, and lack of access to adequate water, sanitation and hygiene facilities at home and school, need to be investigated. Consideration should be made to expand the schistosomiasis and STH control program to community-wide MDA, in conjunction with expanding the school WASH program, to achieve the 2030 targets for schistosomiasis and STH control and elimination.

## Supporting information

S1 Information School water, sanitation and hygiene (WASH) questionnaire. (DOCX)

S2 Information Schistosomiasis prevalence and relative prevalence reduction when compared to the baseline survey for each municipality in Huambo, Uige and Zaire provinces, Angola. (DOCX)

S3 Information Prevalence of schistosomiasis using rapid diagnostic tests and microscopy for each municipality in Huambo, Uige and Zaire Provinces, Angola. (DOCX)

S4 Information Soil-transmitted helminth prevalence and relative prevalence reduction when compared to the baseline survey for each municipality in Huambo, Uige and Zaire provinces, Angola. (DOCX)

S5 Information Results from the school water, sanitation and hygiene (WASH) questionnaire for WASH-supported and WASH-unsupported schools in Huambo, Uige and Zaire provinces, Angola. (DOCX)
