# Supplementary material for: School-based preventive chemotherapy program for schistosomiasis and soil-transmitted helminth control in Angola: 6-year impact assessment
Source: PLoS Negl Trop Dis. 2023 May 17;17(5):e0010849. doi: 10.1371/journal.pntd.0010849 (PMC10228770; doi:10.1371/journal.pntd.0010849)
Supplement: S1 Information — (DOCX) [file pntd.0010849.s001.docx]

**S1 Information.** School water, sanitation and hygiene (WASH) questionnaire

**Date of questionnaire:** _____ / _____ / _____ **Interviewer initials:** ________

**School name:** ______________________________________________________

**School code: ______________________________________________________**

**GPS ID:** ______________________________________________________

**Name of school director completing questionnaire:** ________________________________

**Has written consent been obtained?** Yes ( ) No ( )

*Note to interviewer: only proceed if yes.*

**School data**

Total enrolled students: ____________ No. girls: ___________ No. boys: ___________

No. teachers: ______________ No. female teachers: ________ No. male: teachers: _______

No. grades taught: ____________

Villages that send children to this school: ________________________________________________ __________________________________________________________________________________

**Water supply indicators**

1. What is the main source of water for the school?

*Note to interviewer: read out options, choose ONE option with a tick (√ ). Show the picture sheet of different water sources. Observe the water source if possible.*

| (1) There is no water |  | **🡪** *If no water, go to sanitation section.* | |  |
| --- | --- | --- | --- | --- |
| (2) Piped water into school (inside) |  |  | |  |
| (3) Piped water to yard / plot (outside) |  |  | |  |
| (4) Tubewell or borehole |  |  | |  |
| (5) Protected dug well |  |  | |  |
| (6) Protected spring |  |  | |  |
| (7) Packaged bottle water |  |  | |  |
| (8) Tanker-truck or cart |  |  | |  |
| (9) Unprotected dug well |  |  | |  |
| (10) Unprotected spring |  |  | |  |
| (11) Rainwater |  |  | |  |
| (12) Surface water (e.g, lake, river, stream, pond, canals, irrigation ditches) |  |  | |  |
| (13) Other |  | Specify: |  | |
| (14) Don’t know |  |  | |  |
| (15) No answer |  |  | |  |

2. When is there water available?

| (1) Always throughout the year |  | **🡪** *Go to question 4* |  |
| --- | --- | --- | --- |
| (2) Only in rainy season |  |  |  |
| (3) Don’t know |  |  |  |
| (4) No answer |  |  |  |

3. If water is not available all year round, how often is it not available?

*Note to interviewer: read out options, choose ONE option with a tick (√ ).*

| (1) At least once per day |  |  | |  |
| --- | --- | --- | --- | --- |
| (2) One of two days each week |  |  | |  |
| (3) More than two days each week |  |  | |  |
| (4) More than one week per month |  |  | |  |
| (5) More than one month per year |  |  | |  |
| (6) Other |  | Specify: |  | |
| (7) Don’t know |  |  | |  |
| (8) No answer |  |  | |  |

4. Where is the main water source?

*Note to interviewer: read out options, choose ONE option with a tick (√ ).*

| (1) In the school compound |  |  | |  |
| --- | --- | --- | --- | --- |
| (2) Elsewhere in the village |  |  | |  |
| (3) In a neighbouring village |  |  | |  |
| (4) Other |  | Specify: |  | |
| (5) Don’t know |  |  |  |  |
| (6) No answer |  |  |  |  |

5. Is there someone (a person or a group of people) responsible for fetching water for use in the school?

| (1) Yes |  |  |  |
| --- | --- | --- | --- |
| (2) No |  | **🡪** *Go to question 6.* |  |
| (3) Don’t know |  | **🡪** *Go to question 6.* |  |
| (4) No answer |  | **🡪** *Go to question 6.* |  |

6. Who is responsible for fetching water? __________________________________________

7. What is the distance to fetch water (round trip)?

*Note to interviewer: read out options, choose ONE option with a tick (√ ).*

| (1) Less than 30 min |  |
| --- | --- |
| (2) More than 30 min |  |
| (3) Don’t know |  |
| (4) No answer |  |
| (5) Not applicable |  |

8. Do you store water from this main water source in the school?

| (1) Yes |  |  |  |
| --- | --- | --- | --- |
| (2) No |  | **🡪** *Go to sanitation section.* |  |
| (3) Don’t know |  | **🡪** *Go to sanitation section.* |  |
| (4) No answer |  | **🡪** *Go to sanitation section.* |  |

9. What type of container(s) do you use to store the water?

*Note to interviewer: read out option, show picture sheets to as guide to different container types. Tick (√ ) all items mentioned or observed.*

|  |  | Covered? | | | | | |  |  |
| --- | --- | --- | --- | --- | --- | --- | --- | --- | --- |
| 1. Jerry-can |  | (a) All |  | (b) Some |  | (c) None |  |  |  |
| 1. Basin |  | (a) All |  | (b) Some |  | (c) None |  |  |  |
| 1. Ceramic pot |  | (a) All |  | (b) Some |  | (c) None |  |  |  |
| 1. Other |  | (a) All |  | (b) Some |  | (c) None |  | Specify: |  |
| 1. Don’t know |  |  |  |  |  |  |  |  |  |
| 1. No answer |  |  |  |  |  |  |  |  |  |

10. Do you treat or boil any of the school water?

| (1) Yes |  |  |  |
| --- | --- | --- | --- |
| (2) No |  | **🡪** *Go to sanitation section.* |  |
| (3) Don’t know |  | **🡪** *Go to sanitation section.* |  |
| (4) No answer |  | **🡪** *Go to sanitation section.* |  |

11. For what purposes would you use the treated water for?

*Note to interviewer: read out options and tick (√ ) all items mentioned.*

| (1) Drinking |  |  | |
| --- | --- | --- | --- |
| (2) Hand washing |  |  | |
| (3) Cleaning |  |  | |
| (4) Other |  | Specify: |  |
| (5) Don’t know |  |  |  |
| 1. No answer |  |  |  |

12. What do you treat the water with?

*Note to interviewer: read out options and tick (√ ) all items mentioned.*

| (1) Household bleach |  |  | |  |
| --- | --- | --- | --- | --- |
| (2) Boil |  |  | |  |
| (3) Filter |  |  | |  |
| (4) Other |  | Specify: |  | |
| (5) Don’t know |  |  |  |  |
| (6) No answer |  |  |  |  |

**Sanitation indicators**

*Note to interviewer: questions 12 and 13 require observations to be made by the interviewer.*

13. Are the school grounds free of faeces?

| (1) Yes |  |  |  |
| --- | --- | --- | --- |
| (2) No |  |  |  |
| (3) Could not observe |  |  |  |

14. Are the school grounds free of signs or urine?

| (1) Yes |  |  |  |
| --- | --- | --- | --- |
| (2) No |  |  |  |
| (3) Could not observe |  |  |  |

15. Are there toilets / latrines at school available for students?

| (1) Yes |  | 🡪 *Go to question 15.* |  |
| --- | --- | --- | --- |
| (2) No |  |  |  |
| (3) Don’t know |  | 🡪 *Go to hygiene section.* |  |
| (4) No answer |  | 🡪 *Go to hygiene section.* |  |

16. If there are no toilets / latrines at school available for students, why?

*Note to interviewer: tick (√ ) all items mentioned WITHOUT reading out options. Observations of the toilets / latrines may be required to confirm.*

| (1) There are no toilet / latrine facilities |  | 🡪 *Go to hygiene section* | |  |
| --- | --- | --- | --- | --- |
| (2) There is no water |  |  | |  |
| (3) Toilet / latrine is dirty |  |  | |  |
| (4) Toilet / latrine is broken |  |  | |  |
| (5) Latrine pit is full / overflowing |  |  | |  |
| (6) Toilets / latrines are for teachers only |  |  | |  |
| (7) Other |  | Specify: |  | |
| (8) Don’t know |  |  |  |  |
| (9) No answer |  |  |  |  |

17. Who built the toilet / latrine facilities? _________________________________________

18. When were the toilet / latrine facilities built? ____________________________________

19. When did the toilet / latrine facilities start being used? ____________________________

*Note to interviewer: if toilet / latrine facilities not used enter “Not used”.*

20. Are the toilets / latrines for students functional?

*Note to interviewer: observations of the toilets / latrines may be required to confirm.*

| (1) All |  |  |  |
| --- | --- | --- | --- |
| (2) Some |  |  |  |
| (3) None |  |  |  |
| (4) Don’t know |  |  |  |
| (5) No answer / observation |  |  |  |

21. How many latrines for students are functional? Total:______ Male:______ Female:______

*Note to interviewer: if there are NOT separate facilities for males and females, enter total number then enter “0” for Male and Female,*

22. What kind of toilet / latrine facilities are available?

*Note to interviewer: use picture sheets as guide to different toilet types. Observations of the toilets / latrines may be required to confirm. Tick (√ ) all items mentioned or observed.*

| (1) Flush toilet |  |  |  | |  |  | | | |  |  |
| --- | --- | --- | --- | --- | --- | --- | --- | --- | --- | --- | --- |
| (2) Pit latrine |  | 🡪 | (a) With slab | |  | (i) Do not flush (direct pit) | | | |  |  |
|  |  |  | (b) Without slab | |  | (ii) Flush with water (offset pit) | | | |  |  |
|  |  |  |  | |  | (iii) Flush with water (flush elsewhere) | | | |  |  |
| (3) Composting toilet |  |  |  | |  |  | | | |  |  |
| (4) Bucket latrine |  |  |  | |  |  | | | |  |  |
| (5) Hanging latrine |  |  |  | |  |  | | | |  |  |
| (6) No structure / outdoors |  |  |  | |  |  | | | |  |  |
| (7) Other |  | Specify: | |  | | | | | | | |
| (8) Don’t know |  |  |  | | |  |  |  |  | | |
| (9) No answer |  |  |  | | |  |  |  |  | | |

23. *Note to interviewer: observe the toilet / latrine facilities and tick (√) all relevant observations.*

| (1) Toilet is clean (no urine, faeces, flies) |  |  | |  |  |
| --- | --- | --- | --- | --- | --- |
| (2) Water or other personal cleaning materials are evident |  |  | |  |  |
| (3) There is a hole cover |  |  | |  |  |
| (4) Urine on seat |  |  | |  |  |
| (5) Faeces on seat |  |  | |  |  |
| (6) Urine on floor / walls |  |  | |  |  |
| (7) Faeces on floor/walls |  |  | |  |  |
| (8) Odour |  |  | |  |  |
| (9) Flies present |  |  | |  |  |
| (10) Other |  | Specify: |  | | |
| (11) Could not observe |  |  |  |  |  |

24. Do the toilets / latrines for students have doors?

*Note to interviewer: read out options, observations of the toilets / latrines may be required to confirm. Choose ONE option with a tick (√ ).*

| (1) All |  |  |  |
| --- | --- | --- | --- |
| (2) Some |  |  |  |
| (3) None |  | *.* |  |
| (4) Don’t know |  |  |  |
| (5) No answer / observation |  |  |  |

25. Can the toilets / latrines for students be locked from the inside?

*Note to interviewer: read out options, observations of the toilets / latrines may be required to confirm. Choose ONE option with a tick (√ ).*

| (1) All |  |  |  |
| --- | --- | --- | --- |
| (2) Some |  |  |  |
| (3) None |  | *.* |  |
| (4) Don’t know |  |  |  |
| (5) No answer / observation |  |  |  |

**Hygiene indicators**

26. Are there handwashing facilities?

*Note to interviewer: observations of handwashing facilities may be required to confirm.*

| (1) Yes |  |  |  |
| --- | --- | --- | --- |
| (2) No |  | 🡪 *End questionnaire* |  |
| (3) Don’t know |  | 🡪 *End questionnaire* |  |
| (4) No answer / could not observe |  | 🡪 *End questionnaire* |  |

27. What type of facilities are available for handwashing?

*Note to interviewer: read out options, use picture sheets as guide to different handwashing facilities. Observations of the handwashing facilities may be required to confirm. Tick (√ ) all items mentioned or observed.*

| (1) Sink with tap |  |  |  | |  |  | | | |  |  |
| --- | --- | --- | --- | --- | --- | --- | --- | --- | --- | --- | --- |
| (2) Water tank with tap |  |  |  | |  |  | | | |  |  |
| (3) Tippy tap |  |  |  | |  |  | | | |  |  |
| (4) Bucket / container |  | 🡪 | (a) With tap | |  |  | | | |  |  |
|  |  |  | (b) Without tap | |  |  | | | |  |  |
|  |  |  |  | |  |  | | | |  |  |
| (5) Other |  | Specify: | |  | | | | | | | |
| (6) Don’t know |  |  |  | | |  |  |  |  | | |
| (7) No answer / could not observe |  |  |  | | |  |  |  |  | | |

28. What of the following are available at handwashing facilities?

*Note to interviewer: read out options, observations of the handwashing facilities may be required to confirm. Tick (√ ) all items mentioned or observed.*

| (1) Soap |  |  | |  |  |
| --- | --- | --- | --- | --- | --- |
| (2) Soapy water (prepared solution of detergent in water) |  |  | |  |  |
| (3) Ash |  |  | |  |  |
| (4) Mud |  |  | |  |  |
| (5) Other |  | Specify: |  | | |
| (6) Don’t know |  |  |  |  |  |
| (7) No answer / could not observe |  |  |  |  |  |

29. How many handwashing stations are available for students? ___________________

*Note to interviewer: observations of the handwashing facilities may be required to confirm.*

***This completes the questionnaire. We are grateful for your participation, thank you.***
