# Supplementary material for: School-based preventive chemotherapy program for schistosomiasis and soil-transmitted helminth control in Angola: 6-year impact assessment
Source: PLoS Negl Trop Dis. 2023 May 17;17(5):e0010849. doi: 10.1371/journal.pntd.0010849 (PMC10228770; doi:10.1371/journal.pntd.0010849)
Supplement: S2 Information — (DOCX) [file pntd.0010849.s002.docx]

**S2 Information.** Schistosomiasis prevalence and relative prevalence reduction when compared to the baseline survey for each municipality in Huambo, Uige and Zaire provinces, Angola.

|  | **Impact assessment** | | | | **Prevalence comparison for any schistosomiasis** | |
| --- | --- | --- | --- | --- | --- | --- |
|  | **Schools / Students** | ***S. mansoni*** | ***S. haematobium*** | **Any schistosomiasis** | **Any schistosomiasis at baseline** | **Relative prevalence reduction^a^** |
|  | **N/N** | **% (95%CI)** | **% (95%CI)** | **% (95%CI)** | **% (95%CI)** | **% (95%CI)** |
| **Huambo** |  |  |  |  |  |  |
| Bailundo | 20/600 | 33.0  (25.3, 41.8) | 7.0  (3.8, 12.4) | 37.3  (29.2, 46.3) | 37.1  (27.6, 47.7) | -0.7  (-34.4, 33.0) |
| Caala | 20/600 | 10.7  (6.2, 17.7) | 12.3  (8.3, 18.0) | 20.7  (14.7, 28.3) | 39.0  (29.3, 49.7) | 47.1  (25.8, 68.3) |
| Catchiungo | 20/598 | 10.2  (6.7, 15.3) | 12.0  (6.8, 20.3) | 20.6  (14.1, 29.0) | 42.6  (33.5, 52.2) | 51.7  (32.6, 70.8) |
| Ekunha | 20/597 | 13.2  (7.3, 22.9) | 13.9  (6.0, 29.1) | 22.3  (12.4, 36.8) | 32.6  (16.8, 53.7) | 31.6  (-18.2, 81.3) |
| Huambo | 20/599 | 30.7  (22.6, 40.2) | 9.7  (5.9, 15.6) | 35.9  (27.4, 45.3) | 25.8  (20.2, 32.2) | -39.3  (-84.5, 5.9) |
| Londuimbali | 20/600 | 37.8  (31.9, 44.2) | 4.3  (2.9, 6.6) | 39.7  (33.6, 46.1) | 40.5  (29.1, 53.0) | 2.1  (-29.6, 33.7) |
| Longongjo | 20/600 | 23.3  (17.7, 30.1) | 6.7  (3.4, 12.8) | 28.5  (22.7, 35.1) | 36.5  (25.0, 49.9) | 22.0  (-7.9, 51.9) |
| Mungo | 20/598 | 33.1  (24.8, 42.6) | 7.7  (5.1, 11.5) | 37.1  (28.8, 46.2) | 22.7  (5.9, 57.8) | -63.8  (-194.8, 67.2) |
| Tchicala Tcholohoanga | 21/600 | 15.1  (9.9, 22.5) | 11.2  (7.7, 16.1) | 24.2  (17.2, 32.8) | 40.0  (28.1, 53.2) | 39.6  (14.0, 65.1) |
| Tchinjenje | 20/600 | 19.3  (13.8, 26.4) | 7.8  (4.0, 14.9) | 25.3  (18.0, 34.3) | 40.0  (26.2, 55.6) | 36.7  (7.3, 66.1) |
| Ukuma | 20/599 | 27.2  (19.4, 36.8) | 10.4  (5.8, 17.7) | 33.6  (24.2, 44.4) | 48.6  (33.8, 63.6) | 30.9  (3.4, 58.4) |
| **Total** | **221/6,591** | **23.1**  **(20.8, 25.5)** | **9.4**  **(7.9, 11.1)** | **29.6**  **(27.1, 32.2)** | **36.4**  **(33.1, 39.8)** | **18.8**  **(8.6, 29.0)** |
| **Uige** |  |  |  |  |  |  |
| Ambuila | 15/450 | 30.9  (18.2, 47.3) | 1.8  (0.6, 5.3) | 32.0  (19.3, 48.1) | 24.2  (11.4, 44.3) | -32.1  (-125.0, 60.7) |
| Bembe | 17/510 | 30.2  (16.3, 49.0) | 0.4  (0.1, 1.7) | 30.4  (16.6, 49.0) | 35.6  (22.4, 51.3) | 14.5  (-38.7, 67.7) |
| Buengas | 15/450 | 33.8  (22.2, 47.7) | 1.3  (0.4, 4.1) | 34.9  (23.2, 48.7) | 6.7  (3.9, 11.1) | -422.5  (-729.9, -115.2) |
| Bungo | 17/495 | 31.6  (19.1, 47.4) | 20.0  (11.9, 31.7) | 46.6  (33.6, 60.0) | 12.6  (8.5, 18.2) | -269.8  (-428.9, -110.7) |
| Cangola | 16/494 | 10.9  (6.3, 18.4) | 6.5  (3.2, 12.6) | 17.0  (11.8, 23.9) | 9.1  (5.4, 14.8) | -87.4  (-192.7, 17.9) |
| Damba | 17/509 | 37.7  (26.9, 49.8) | 14.4  (9.4, 21.4) | 47.5  (37.8, 57.4) | 9.3  (5.7, 14.7) | -413.5  (-660.4, -166.6) |
| Maquela do Zombo | 17/508 | 47.8  (33.9, 62.1) | 9.1  (5.1, 15.7) | 53.9  (40.9, 66.4) | 9.4  (6.1, 14.1) | -475.0  (-730.9, -219.1) |
| Milunga | 16/479 | 26.5  (16.7, 39.3) | 2.1  (1.0, 4.1) | 27.8 (18.3, 39.8) | 6.7  (3.0, 14.4) | -313.6  (-624.2, 2.9) |
| Mucaba | 17/494 | 9.1  (3.8, 20.1) | 20.6  (13.3, 30.7) | 28.7  (20.2, 39.1) | 13.3  (7.4, 22.7) | -115.9  (-241.2, 9.4) |
| Negage | 18/552 | 30.8  (21.1, 42.6) | 16.5  (9.7, 26.6) | 40.2  (30.8, 50.5) | 20.0  (15.4, 25.4) | -101.5  (-166.4, -36.5) |
| Puri | 17/505 | 27.2  (17.6, 39.5) | 11.3  (6.4, 19.1) | 36.3  (26.6, 47.2) | 11.3  (6.0, 20.3) | -22.0  (-405.2, -35.6) |
| Quimbele | 16/480 | 23.3  (13.8, 36.7) | 2.7  (1.4, 5.1) | 25.2  (15.3, 38.6) | 6.5  (4.4, 9.7) | -285.6  (-505.8, -65.3) |
| Quitexe | 17/510 | 20.8  (11.6, 34.4) | 5.5  (1.6, 16.9) | 25.3  (15.3, 39.0) | 15.0  (5.1, 36.9) | -69.0  (-211.7, 73.8) |
| Sanza Pombo | 18/544 | 22.2  (12.0, 37.6) | 3.9  (1.5, 9.3) | 25.6  (14.9, 40.1) | 9.3  (6.7, 12.8) | -173.7  (-323.8, -23.6) |
| Songo | 16/480 | 52.5  (40.0, 64.7) | 4.6  (1.7, 11.9) | 53.8  (41.4, 65.7) | 77.6  (59.7, 89.0) | 30.8  (12.1, 49.4) |
| Uige | 17/508 | 39.6  (27.5, 53.0) | 3.0  (1.4, 5.9) | 41.1  (29.2, 54.2) | 45.4  (31.2, 60.4) | 9.4  (-28.3, 47.1) |
| **Total** | **266/7,963** | **29.6**  **(26.6, 32.8)** | **7.8**  **(6.5, 9.4)** | **35.4**  **(32.5, 38.5)** | **18.4**  **(15.8, 21.4)** | **-92.3**  **(-126.2, -58.3)** |
| **Zaire** |  |  |  |  |  |  |
| Kuimba | 19/571 | 27.5  (19.2, 37.6) | 1.1  (0.2, 4.8) | 27.8  (19.7, 37.7) | 30.6  (20.5, 42.8) | 8.9  (-31.1, 48.9) |
| Mbanza Kongo | 20/598 | 24.4  (14.3, 38.4) | 27.5  (14.3, 46.3) | 41.2  (25.3, 59.1) | 39.0  (23.0, 57.8) | -5.7  (-65.7, 54.4) |
| Noqui | 18/540 | 16.5  (9.0, 28.3) | 20.2  (10.0, 36.7) | 27.3  (15.1, 44.1) | 28.1  (13.3, 50.1) | 3.1  (-69.0, 75.2) |
| Nzeto | 18/540 | 10.4  (6.1, 17.1) | 25.7  (14.4, 41.7) | 30.9  (17.9, 47.9) | 21.3  (6.1, 53.2) | -45.0  (-169.8, 79.8) |
| Soyo | 18/540 | 20.9  (15.9, 27.0) | 3.0  (1.5, 5.7) | 23.0  (18.0, 28.8) | 10.3  (3.2, 28.3) | -122.8  (-338.6, 92.9) |
| Tomboco | 19/541 | 17.4  (12.0, 24.5) | 0.7  (0.3, 1.9) | 17.9  (12.6, 24.8) | 6.7  (2.5, 16.3) | -168.9  (-367.1, 29.2) |
| **Total** | **112/3,326** | **19.7**  **(16.5, 23.3)** | **13.2**  **(9.2, 18.4)** | **28.2**  **(23.6, 33.4)** | **24.8**  **(18.9, 31.7)** | **-14.0**  **(-48.6, 20.6)** |

Prevalence calculations adjusted for clustering at school level. ^a^Relative prevalence reduction = (2014 prevalence – 2021 prevalence) / 2014 prevalence; negative values represent a relative increase in prevalence and positive values represent a relative reduction in prevalence. N = number surveyed. RDT = rapid diagnostic test.
