# Supplementary material for: School-based preventive chemotherapy program for schistosomiasis and soil-transmitted helminth control in Angola: 6-year impact assessment
Source: PLoS Negl Trop Dis. 2023 May 17;17(5):e0010849. doi: 10.1371/journal.pntd.0010849 (PMC10228770; doi:10.1371/journal.pntd.0010849)
Supplement: S3 Information — (DOCX) [file pntd.0010849.s003.docx]

**S3 Information.** Prevalence of schistosomiasis using rapid diagnostic tests and microscopy for each municipality in Huambo, Uige and Zaire provinces, Angola.

|  | ***Schistosoma mansoni*** | | | ***Schistosoma haematobium*** | | | **Any schistosomiasis** | | |
| --- | --- | --- | --- | --- | --- | --- | --- | --- | --- |
|  | **RDT** | | **Microscopy** | **RDT** | | **Microscopy** | **RDT** | | **Microscopy** |
|  | **Trace pos**  **% (95%CI)** | **Trace neg**  **% (95%CI)** | **% (95%CI)** | **Trace pos**  **% (95%CI)** | **Trace neg**  **% (95%CI)** | **% (95%CI)** | **Trace pos**  **% (95%CI)** | **Trace neg**  **% (95%CI)** | **% (95%CI)** |
| **Huambo** |  |  |  |  |  |  |  |  |  |
| Bailundo | 33.0  (25.3, 41.8) | 10.3  (7.0, 15.0) | 0 | 7.0  (3.8, 12.4) | 1.8  (0.7, 4.4) | 0.4  (0.04, 3.6) | 37.3  (29.2, 46.3) | 11.8  (8.1, 17.0) | 0.4  (0.04, 3.6) |
| Caala | 10.7  (6.2, 17.7) | 3.3  (1.5, 7.4) | 0.4  (0.04, 3.6) | 12.3  (8.3, 18.0) | 8.0  (5.1, 12.3) | 6.3  (2.0, 17.9) | 20.7  (14.7, 28.3) | 10.5  (6.7, 16.1) | 6.7  (2.3, 17.8) |
| Catchiungo | 10.2  (6.7, 15.3) | 2.8  (1.3, 6.3) | 0 | 12.0  (6.8, 20.3) | 1.5  (0.6, 3.5) | 1.1  (0.2, 5.5) | 20.6  (14.1, 29.0) | 3.8  (1.9, 7.7) | 1.3  (0.2, 6.4) |
| Ekunha | 13.2  (7.3, 22.9) | 4.7  (1.6, 12.7) | 0.8  (0.2, 3.8) | 13.9  (6.0, 29.1) | 2.8  (0.5, 14.7) | 0.5  (0.04, 5.3) | 22.3  (12.4, 36.8) | 7.4  (2.2, 22.3) | 1.4  (0.4, 5.4) |
| Huambo | 30.7  (22.6, 40.2) | 8.0  (4.7, 13.4) | 0.3  (0.03, 3.2) | 9.7  (5.9, 15.6) | 2.7  (0.9, 7.9) | 7.9  (2.1, 25.6) | 35.9  (27.4, 45.3) | 10.4  (6.5, 16.1) | 8.3  (2.4, 24.9) |
| Londuimbali | 37.8  (31.9, 44.2) | 8.2  (5.5, 11.9) | 0 | 4.3  (2.9, 6.6) | 1.0  (0.5, 2.1) | 0.4  (0.04, 3.6) | 39.7  (33.6, 46.1) | 9.0  (6.4, 12.5) | 0.4  (0.04, 3.6) |
| Longongjo | 23.3  (17.7, 30.1) | 4.5  (2.3, 8.7) | 0 | 6.7  (3.4, 12.8) | 3.0  (1.1, 8.2) | 4.1  (0.7, 19.3) | 28.5  (22.7, 35.1) | 7.5  (4.6, 11.9) | 4.1  (0.7, 19.3) |
| Mungo | 33.1  (24.8, 42.6) | 10.2  (5.5, 18.1) | 0.7  (0.2, 3.4) | 7.7  (5.1, 11.5) | 1.5  (0.6, 3.5) | 0 | 37.1  (28.8, 46.2) | 11.0  (6.3, 18.7) | 0.7  (0.2, 3.4) |
| Tchicala  Tcholohoanga | 15.1  (9.9, 22.5) | 4.2  (1.9, 9.0) | 1.1  (0.1, 10.4) | 11.2  (7.7, 16.1) | 0.7  (0.2, 2.4) | 1.1  (0.1, 10.6) | 24.2  (17.2, 32.8) | 4.8  (2.3, 9.8) | 2.4  (0.4, 14.1) |
| Tchinjenje | 19.3  (13.8, 26.4) | 4.8  (2.5, 9.2) | 0.4  (0.04, 3.6) | 7.8  (4.0, 14.9) | 2.2  (0.9, 5.0) | 0.4  (0.04, 4.3) | 25.3  (18.0, 34.3) | 7.0  (4.3, 11.2) | 0.8  (0.08, 8.4) |
| Ukuma | 27.2  (19.4, 36.8) | 5.3  (3.5, 8.5) | 0.4  (0.04, 3.6) | 10.4  (5.8, 17.7) | 5.5  (2.2, 13.0) | 0.7  (0.2, 3.4) | 33.6  (24.2, 44.4) | 9.8  (5.8, 16.2) | 1.2  (0.3, 4.6) |
| **Total** | **23.1**  **(20.8, 25.5)** | **6.1**  **(5.1, 7.2)** | **0.4**  **(0.2, 0.8)** | **9.4**  **(7.9, 11.1)** | **2.8**  **(2.1, 3.8)** | **2.1**  **(1.2, 3.6)** | **29.6**  **(27.1, 32.2)** | **8.5**  **(7.2, 9.9)** | **2.5**  **(1.5, 4.1)** |
| **Uige** |  |  |  |  |  |  |  |  |  |
| Ambuila | 30.9  (18.2, 47.3) | 14.2  (5.1, 33.9) | 3.3  (0.3, 31.1) | 1.8  (0.6, 5.3) | 0.2  (0.03, 1.9) | 1.1  (0.02, 43.6) | 32.0  (19.3, 48.1) | 16.4  (7.2, 33.2) | 4.4  (0.8, 20.5) |
| Bembe | 30.2  (16.3, 49.0) | 16.9  (7.4, 33.9) | 33.3  (0, 100) | 0.4  (0.1, 1.7) | 0.4  (0.1, 1.7) | 0 | 30.4  (16.6, 49.0) | 17.1  (7.6, 33.9) | 33.3  (0, 100) |
| Buengas | 33.8  (22.2, 47.7) | 18.9  (10.9, 30.6) | 0.8  (0.03, 17.2) | 1.3  (0.4, 4.1) | 0 | 0 | 34.9  (23.2, 48.7) | 18.9  (10.9, 30.6) | 0.8  (0.03, 17.2) |
| Bungo | 31.6  (19.1, 47.4) | 15.6  (6.6, 32.4) | 14.4  (0.1, 96.3) | 20.0  (11.9, 31.7) | 10.9  (5.3, 21.2) | 0 | 46.6  (33.6, 60.0) | 25.9  (15.3, 40.3) | 14.4  (0.1, 96.3) |
| Cangola | 10.9  (6.3, 18.4) | 5.5  (3.3, 8.9) | 0 | 6.5  (3.2, 12.6) | 2.4  (0.9, 6.4) | 0 | 17.0  (11.8, 23.9) | 7.9  (5.5, 11.2) | 0 |
| Damba | 37.7  (26.9, 49.8) | 10.7  (6.8, 16.3) | 0 | 14.4  (9.4, 21.4) | 6.3  (2.5, 14.9) | 0 | 47.5  (37.8, 57.4) | 16.8  (11.9, 23.2) | 0 |
| Maquela  do Zombo | 47.8  (33.9, 62.1) | 21.5  (11.7, 36.0) | 3.3  (0.04, 74.7) | 9.1  (5.1, 15.7) | 3.7  (1.3, 10.1) | 1.1  (0.02, 43.6) | 53.9  (40.9, 66.4) | 25.0  (15.6, 37.6) | 4.4  (0.3, 37.8) |
| Milunga | 26.5  (16.7, 39.3) | 17.1  (11.7, 24.4) | 0 | 2.1  (1.0, 4.1) | 0.8  (0.3, 2.2) | 0 | 27.8 (18.3, 39.8) | 18.0  (12.7, 24.8) | 0 |
| Mucaba | 9.1  (3.8, 20.1) | 3.2  (1.8, 5.9) | 8.9  (0.5, 65.1) | 20.6  (13.3, 30.7) | 3.0  (1.5, 6.1) | 0 | 28.7  (20.2, 39.1) | 6.1  (4.2, 8.6) | 8.9  (0.5, 65.1) |
| Negage | 30.8  (21.1, 42.6) | 8.9  (3.6, 20.4) | 0 | 16.5  (9.7, 26.6) | 3.8  (1.4, 10.0) | 0 | 40.2  (30.8, 50.5) | 12.0  (5.8, 23.0) | 0 |
| Puri | 27.2  (17.6, 39.5) | 10.1  (5.2, 18.8) | 3.3  (0.3, 31.1) | 11.3  (6.4, 19.1) | 4.2  (1.9, 9.1) | 1.1  (0.01, 46.6) | 36.3  (26.6, 47.2) | 13.9  (8.8, 21.3) | 4.4  (0.5, 30.6) |
| Quimbele | 23.3  (13.8, 36.7) | 12.9  (8.0, 20.1) | 0 | 2.7  (1.4, 5.1) | 0.6  (0.1, 2.9) | 0 | 25.2  (15.3, 38.6) | 13.3  (8.3, 20.8) | 0 |
| Quitexe | 20.8  (11.6, 34.4) | 9.6  (3.6, 23.1) | 6.7  (0.5, 50.6) | 5.5  (1.6, 16.9) | 1.4  (0.4, 5.1) | 0 | 25.3  (15.3, 39.0) | 11.0  (4.6, 24.0) | 6.7  (0.5, 50.6) |
| Sanza Pombo | 22.2  (12.0, 37.6) | 12.7  (6.5, 23.3) | 0 | 3.9  (1.5, 9.3) | 0.9  (0.4, 2.1) | 0 | 25.6  (14.9, 40.1) | 13.4  (7.0, 24.2) | 0 |
| Songo | 52.5  (40.0, 64.7) | 36.9  (24.7, 50.9) | 12.2  (1.3, 59.5) | 4.6  (1.7, 11.9) | 1.5  (0.2, 8.7) | 0 | 53.8  (41.4, 65.7) | 37.3  (25.0, 51.5) | 12.2  (1.3, 59.5) |
| Uige | 39.6  (27.5, 53.0) | 18.5  (10.8, 29.9) | 3.3  (0.04, 74.7) | 3.0  (1.4, 5.9) | 0.8  (0.2, 3.3) | 0 | 41.1  (29.2, 54.2) | 19.3  (11.7, 30.2) | 3.3  (0.04, 74.7) |
| **Total** | **29.6**  **(26.6, 32.8)** | **14.5**  **(12.3, 16.9)** | **5.1**  **(2.5, 10.2)** | **7.8**  **(6.5, 9.4)** | **2.6**  **(1.9, 3.5)** | **0.2**  **(0.06, 0.7)** | **35.4**  **(32.5, 38.5)** | **16.8**  **(14.7, 19.2)** | **5.4**  **(2.7, 10.4)** |
| **Zaire** |  |  |  |  |  |  |  |  |  |
| Kuimba | 27.5  (19.2, 37.6) | 3.2  (1.6, 6.0) | 0.3  (0.03, 2.4) | 1.1  (0.2, 4.8) | 0.4  (0.04, 2.8) | 0.8  (0.09, 6.2) | 27.8  (19.7, 37.7) | 3.2  (1.6, 6.0) | 1.1  (0.2, 6.9) |
| Mbanza Kongo | 24.4  (14.3, 38.4) | 16.3  (8.3, 29.6) | 4.2  (0.6, 24.6) | 27.5  (14.3, 46.3) | 20.5  (10.5, 36.0) | 0.7  (0.2, 2.1) | 41.2  (25.3, 59.1) | 32.3  (19.9, 47.8) | 4.9  (0.9, 22.7) |
| Noqui | 16.5  (9.0, 28.3) | 5.4  (3.2, 9.0) | 0.3  (0.03, 2.2) | 20.2  (10.0, 36.7) | 11.7  (5.9, 21.8) | 0 | 27.3  (15.1, 44.1) | 15.2  (8.7, 25.3) | 0.3  (0.03, 2.3) |
| Nzeto | 10.4  (6.1, 17.1) | 3.3  (1.6, 6.8) | 0.3  (0.03, 2.5) | 25.7  (14.4, 41.7) | 17.0  (9.1, 29.6) | 2.2  (0.3, 14.0) | 30.9  (17.9, 47.9) | 19.1  (10.7, 31.6) | 2.5  (0.4, 12.8) |
| Soyo | 20.9  (15.9, 27.0) | 2.2  (1.0, 4.9) | 0 | 3.0  (1.5, 5.7) | 1.7  (0.7, 3.9) | 0 | 23.0  (18.0, 28.8) | 3.7  (2.0, 6.8) | 0 |
| Tomboco | 17.4  (12.0, 24.5) | 0.4  (0.09, 1.5) | 0 | 0.7  (0.3, 1.9) | 0.2  (0.02, 1.5) | 0.8  (0.2, 3.6) | 17.9  (12.6, 24.8) | 0.6  (0.2, 1.7) | 0.8  (0.2, 3.8) |
| **Total** | **19.7**  **(16.5, 23.3)** | **5.3**  **(3.5, 7.9)** | **0.9**  **(0.2, 4.2)** | **13.2**  **(9.2, 18.4)** | **8.7**  **(5.9, 12.5)** | **0.7**  **(0.3, 1.8)** | **28.2**  **(23.6, 33.4)** | **12.6**  **(9.2, 16.9)** | **1.7**  **(0.6, 4.4)** |

Prevalence calculations adjusted for clustering at school level. N = number surveyed. Trace pos = trace readings considered positive. Trace neg = trace readings considered negative. RDT = rapid diagnostic test.
