# Supplementary material for: School-based preventive chemotherapy program for schistosomiasis and soil-transmitted helminth control in Angola: 6-year impact assessment
Source: PLoS Negl Trop Dis. 2023 May 17;17(5):e0010849. doi: 10.1371/journal.pntd.0010849 (PMC10228770; doi:10.1371/journal.pntd.0010849)
Supplement: S4 Information — (DOCX) [file pntd.0010849.s004.docx]

**S4 Information.** Soil-transmitted helminth infections prevalence and relative prevalence reduction when compared to the baseline survey for each municipality in Huambo, Uige and Zaire provinces, Angola.

|  | **Impact assessment** | | | | | **Prevalence comparison** | |
| --- | --- | --- | --- | --- | --- | --- | --- |
|  | **Schools / students** | ***Ascaris lumbricoides*** | **Hookworm** | ***Trichuris trichiura*** | **Any STH** | **Any STH at baseline** | **Relative prevalence reduction for any STH^a^** |
|  | **N/N** | **% (95%CI)** | **% (95%CI)** | **% (95%CI)** | **% (95%CI)** | **% (95%CI)** | **% (95%CI)** |
| **Huambo** |  |  |  |  |  |  |  |
| Bailundo | 9/270 | 7.4  (2.2, 22.5) | 17.4  (10.0, 28.5) | 7.0  (3.6, 13.4) | 25.2  (15.4, 38.3) | 3.3  (1.4, 7.9) | -655.6  (-1,176.9, -134.2) |
| Caala | 9/270 | 19.6  (8.2, 40.0) | 0.4  (0.04, 3.6) | 0.4  (0.04, 3.6) | 20.0  (8.5, 40.2) | 10.9  (5.2, 21.3) | -83.5  (-241.9, 74.9) |
| Catchiungo | 9/270 | 3.7  (1.5, 8.7) | 0 | 0.4  (0.04, 3.6) | 4.1  (1.9, 8.5) | 4.7  (2.1, 10.1) | 12.7  (-58.5, 83.9) |
| Ekunha | 9/270 | 14.4  (6.9, 27.6) | 0 | 3.0  (1.3, 6.5) | 16.7  (8.8, 29.3) | 16.7  (0.3, 92.9) | 0  (-144.3, 144.3) |
| Huambo | 10/298 | 27.5  (13.7, 47.6) | 0.7  (0.1, 3.0) | 1.3  (0.4, 4.7) | 29.2  (15.4, 48.4) | 28.8  (13.3, 51.6) | -1.4  (-79.4, 76.6) |
| Londuimbali | 9/270 | 8.5  (5.5, 12.9) | 7.8  (3.1, 18.3) | 1.5  (0.4, 5.1) | 16.7  (10.0, 26.4) | 0.8  (0.03, 17.2) | -1,849.6  (-5,525.2, 1,725.2) |
| Longongjo | 9/270 | 10.0  (5.0, 18.9) | 1.1  (0.2, 5.5) | 3.0  (1.2, 7.2) | 12.6  (7.0, 21.7) | 20.0  (1.0, 85.8) | 37.0  (-72.3, 146.3) |
| Mungo | 9/270 | 8.9  (3.5, 20.8) | 1.9  (0.5, 6.1) | 3.0  (0.7, 12.1) | 13.0  (7.1, 22.5) | 3.3  (0, 100) | -288.9  (-479.4, 98.4) |
| Tchicala Tcholohoanga | 9/270 | 15.6  (8.6, 26.6) | 2.2  (0.5, 9.8) | 1.5  (0.3, 8.2) | 17.8  (10.4, 28.7) | 3.3  (0.3, 26.1) | -433.3  (-1,137.1, 270.4) |
| Tchinjenje | 9/270 | 4.4  (0.8, 21.3) | 1.5  (0.3, 6.6) | 3.3  (1.7, 6.4) | 8.9  (3.7, 19.5) | 3.3  (0, 100) | -166.7  (-596.4, 263.1) |
| Ucuma | 9/270 | 9.6  (5.4, 16.7) | 5.2  (1.7, 14.6) | 1.5  (0.4, 5.1) | 14.4  (9.4, 21.5) | 8.9  (0.5, 65.1) | -62.5  (-241.4, 116.4) |
| **Total** | **100/2,998** | **11.9**  **(9.4, 15.0)** | **3.4**  **(2.3, 5.2)** | **2.3**  **(1.7, 3.2)** | **16.3**  **(13.7, 19.4)** | **12.7**  **(7.8, 20.1)** | **-28.4**  **(-92.1, 35.2)** |
| **Uige** |  |  |  |  |  |  |  |
| Ambuila | 3/90 | 70.0  (17.5, 96.3) | 1.1  (0.01, 46.6) | 13.3  (0.6, 80.4) | 71.1  (18.8, 96.3) | 35.0  (0.5, 98.3) | -103.2  (-197.7, 8.6) |
| Bembe | 2/60 | 20.0  (0, 100) | 1.7  (0, 100) | 6.7  (0.01, 98.5) | 26.7  (0.05, 99.6) | 6.7  (0.5, 50.6) | -300.0  (-774.1, 174.1) |
| Buengas | 4/120 | 65.0  (17.7, 94.1) | 1.7  (0.3, 9.9) | 31.7  (2.1, 90.8) | 70.8  (17.7, 96.5) | 76.7  (23.0, 97.3) | 7.6  (-39.8, 55.1) |
| Bungo | 3/90 | 55.6  (42.8, 67.6) | 1.1  (0.01, 46.6) | 2.2  (0.3, 17.0) | 57.8  (40.3, 73.5) | 72.2  (17.8, 96.9) | 20.0  (-4.5, 44.5) |
| Cangola | 3/90 | 73.3  (37.4, 92.7) | 8.9  (0.7, 56.1) | 0 | 75.6  (42.2, 92.9) | 65.8  (22.9, 92.6) | -14.8  (-60.0, 30.5) |
| Damba | 3/92 | 72.2  (3.7, 99.4) | 0 | 2.2  (0.03, 64.9) | 73.3  (4.4, 99.4) | 74.2  (35.2, 93.8) | 1.1  (-48.6, 50.8) |
| Maquela do Zombo | 3/90 | 60.0  (1.1, 99.5) | 0 | 6.7  (0.5, 50.6) | 60.0  (1.1, 99.5) | 76.0 (34.8, 95.0) | 21.1  (-44.5, 86.6) |
| Milunga | 1/30 | 80.0  (0, 100) | 3.3  (0, 100) | 0 | 80.0  (0, 100) | 55.0  (0, 100) | -45.5  (-172.6, 81.7) |
| Mucaba | 3/90 | 94.4  (87.2, 97.7) | 1.1  (0.01, 46.6) | 2.2  (0.03, 64.9) | 95.6  (87.5, 98.5) | 76.7  (22.1, 97.4) | -24.6  (-53.8, 4.5) |
| Negage | 4/120 | 66.7  (49.8, 80.2) | 15.8  (0.4, 89.2) | 3.3  (0.1, 48.1) | 68.3  (50.2, 82.2) | 73.3  (30.3, 94.6) | 6.8  (-15.7, 29.3) |
| Puri | 3/90 | 28.9  (12.8, 52.8) | 0 | 0 | 28.9  (12.8, 52.8) | 58.3  (9.3, 95.0) | 50.5  (34.2, 66.7) |
| Quimbele | 3/90 | 74.4  (15.1, 98.0) | 4.4  (0.05, 80.8) | 23.3  (1.1, 89.6) | 78.9  (8.0, 99.4) | 84.7  (74.0, 91.4) | 6.8  (-23.3, 37.0) |
| Quitexe | 3/90 | 84.4  (79.0, 88.7) | 3.3  (0, 100) | 10.0  (4.2, 21.8) | 85.6  (80.1, 89.7) | 91.7  (0.3, 100) | 2.0  (2.0, 2.0) |
| Sanza Pombo | 3/95 | 47.8  (3.0, 96.4) | 11.1  (0.4, 80.4) | 0 | 51.1  (2.5, 97.7) | 48.7  (30.4, 67.3) | -5.0  (-85.5, 75.5) |
| Songo | 3/90 | 48.9  (17.7, 81.0) | 0 | 7.8  (0.8, 48.3) | 52.2  (28.4, 75.1) | 63.3  (15.7, 94.1) | 17.5  (-14.4, 49.4) |
| Uige | 3/99 | 60.6  (9.4, 95.8) | 0 | 0 | 60.6  (9.4, 95.8) | 27.7  (9.0, 59.7) | -119.0  (-326.1, 88.1) |
| **Total** | **46/1,419** | **62.9**  **(55.0, 70.2)** | **3.6**  **(1.5, 8.6)** | **7.5**  **(4.0, 13.7)** | **65.1**  **(57.2, 72.3)** | **58.8**  **(50.5, 66.6)** | **-10.7**  **(-30.2, 8.8)** |
| **Zaire** |  |  |  |  |  |  |  |
| Kuimba | 12/366 | 47.5  (31.7, 63.9) | 2.5  (0.5, 13.0) | 2.7  (1.1, 6.7) | 49.2  (33.2, 65.2) | 20.0  (1.7, 77.9) | -145.9  (-240.2, -51.6) |
| Mbanza kongo | 15/450 | 33.1  (19.6, 50.2) | 6.7  (1.6, 23.6) | 2.4  (1.0, 6.1) | 36.4  (22.0, 53.9) | 11.7  (0.03, 98.4) | -212.4  (-442.9, 18.1) |
| Noqui | 13/390 | 10.8  (3.4, 29.1) | 2.1  (0.7, 6.1) | 3.8  (1.7, 8.6) | 14.4  (6.3, 29.4) | 25.0  (0, 100) | 42.6  (-39.3, 124.4) |
| Nzeto | 12/359 | 3.3  (1.4, 7.7) | 1.1  (0.3, 3.8) | 3.1  (1.1, 8.2) | 7.2  (4.3, 12.0) | 6.7  (0, 100) | -8.6  (-58.2, 40.9) |
| Soyo | 14/419 | 28.9  (18.8, 41.6) | 3.6  (0.5, 20.4) | 19.6  (12.8, 28.7) | 38.4  (25.6, 53.1) | 38.9  (5.9, 86.6) | 1.2  (-61.6, 64.0) |
| Tomboco | 14/420 | 16.9  (9.4, 28.6) | 1.7  (0.3, 7.6) | 5.7  (3.0, 10.7) | 21.7  (13.2, 33.5) | 20.0  (0, 100) | -8.3  (-54.5, 37.8) |
| **Total** | **80/2,404** | **23.7**  **(18.6, 29.6)** | **3.0**  **(1.5, 5.9)** | **6.4**  **(4.6, 8.8)** | **28.2**  **(22.8, 34.3)** | **23.3**  **(13.3, 37.6)** | **-20.9**  **(-79.5, 37.8)** |

Prevalence calculations adjusted for clustering at school level. ^a^Relative prevalence reduction = (2014 prevalence – 2021 prevalence) / 2014 prevalence; negative point estimates represent a relative increase in prevalence and positive point estimates represent a relative reduction in prevalence. CI = confidence interval. STH = soil-transmitted helminth.
