# Supplementary material for: School-based preventive chemotherapy program for schistosomiasis and soil-transmitted helminth control in Angola: 6-year impact assessment
Source: PLoS Negl Trop Dis. 2023 May 17;17(5):e0010849. doi: 10.1371/journal.pntd.0010849 (PMC10228770; doi:10.1371/journal.pntd.0010849)
Supplement: S5 Information — (DOCX) [file pntd.0010849.s005.docx]

**S5 Information.** Results from the school water, sanitation and hygiene (WASH) questionnaire for WASH-supported and WASH-unsupported schools in Huambo, Uige and Zaire provinces, Angola.

|  | **Overall** | **WASH supported** | **WASH unsupported** | **P-value^a^** |
| --- | --- | --- | --- | --- |
| **Schools (N)** | 589 | 178 | 411 |  |
| **Students per school, mean (range)** | 593 (2-4,467) | 508 (2-4,467) | 554 (30-4,195) |  |
| **Setting, %(n)** |  |  |  |  |
| **Rural** | 68.9 (405) | 72.5 (129) | 67.2 (276) |  |
| **Urban** | 31.1 (183) | 27.5 (49) | 32.6 (134) |  |
| **Missing** | 0.2 (1) | 0 | 0.2 (1) |  |
| **Water indicators** | | | | |
| **Water availability, %(n)** |  |  |  |  |
| All the time | 75.0 (442) | 79.8 (142) | 73.0 (300) | 0.08 |
| During wet season only | 8.0 (47) | 6.7 (12) | 8.5 (35) | 0.47 |
| Never | 6.3 (37) | 5.6 (10) | 6.6 (27) | 0.66 |
| Other | 3.4 (20) | 3.4 (6) | 3.4 (14) | 0.98 |
| Don’t know / no reply | 7.3 (43) | 4.5 (8) | 8.5 (35) | 0.09 |
| **Water source, %(n)** |  |  |  |  |
| In school complex | 15.1 (89) | 19.1 (34) | 13.4 (55) | 0.08 |
| Elsewhere in village | 67.2 (396) | 66.3 (118) | 67.6 (278) | 0.75 |
| In neighbouring village | 9.0 (53) | 8.4 (15) | 9.3 (38) | 0.75 |
| Other | 2.6 (15) | 3.9 (7) | 2.0 (8) | 0.16 |
| Don’t know / no reply | 6.5 (38) | 2.3 (4) | 8.3 (34) | 0.005 |
| **Type of water source, %(n)** |  |  |  |  |
| Piped water into school | 4.8 (28) | 3.4 (6) | 5.4 (22) | 0.30 |
| Piped water to yard / plot outside of school | 3.9 (23) | 11.2 (20) | 0.7 (3) | **<0.001** |
| Protected well | 12.7 (75) | 17.4 (31) | 10.7 (44) | **0.03** |
| Unprotected well | 9.2 (54) | 10.7 (19) | 8.5 (35) | 0.41 |
| Tubewell or borehole | 7.8 (46) | 7.3 (13) | 8.0 (33) | 0.76 |
| Tanker truck | 1.9 (11) | 1.7 (3) | 2.0 (8) | 1.0 |
| Protected spring | 1.7 (10) | 1.7 (3) | 1.7 (7) | 1.0 |
| Unprotected spring | 6.1 (36) | 1.1 (2) | 8.3 (34) | **<0.001** |
| Surface water | 37.5 (221) | 33.7 (60) | 39.2 (161) | 0.21 |
| Other | 4.1 (24) | 3.9 (7) | 4.1 (17) | 0.91 |
| No water source | 11.9 (70) | 9.6 (17) | 12.9 (53) | 0.25 |
| Don’t know / no reply | 1.7 (10) | 0.6 (1) | 2.2 (9) | 0.30 |
| **Someone responsible for fetching water, %(n)** |  |  |  | **0.005** |
| Yes | 73.2 (431) | 78.7 (140) | 70.8 (291) |  |
| No | 20.7 (122) | 19.7 (35) | 21.2 (87) |  |
| Don’t know / no reply | 6.1 (36) | 1.7 (3) | 8.0 (33) |  |
| **Distance to water, %(n)** |  |  |  | 0.05 |
| Less than 30 minutes | 56.5 (333) | 60.1 (107) | 55.0 (226) |  |
| More than 30 minutes | 32.4 (191) | 30.9 (55) | 33.1 (136) |  |
| Don’t know / no reply | 7.1 (42) | 3.4 (6) | 8.8 (36) |  |
| Not applicable | 3.9 (23) | 5.6 (10) | 3.2 (13) |  |
| **Water stored, %(n)** |  |  |  | **0.01** |
| Yes | 80.3 (473) | 87.1 (155) | 77.4 (318) |  |
| No | 16.3 (96) | 11.8 (21) | 18.3 (75) |  |
| Don’t know / no reply | 3.4 (20) | 1.1 (2) | 4.4 (18) |  |
| **Treat/boil water, %(n)** |  |  |  | **0.002** |
| Yes | 81.8 (482) | 90.5 (161) | 78.1 (321) |  |
| No | 13.9 (82) | 7.9 (14) | 16.6 (68) |  |
| Don’t know / no reply | 4.2 (25) | 1.7 (3) | 5.4 (22) |  |
| **Agent for treating water, %(n)** |  |  |  |  |
| Household bleach | 80.7 (475) | 89.9 (160) | 76.7 (315) | **<0.001** |
| Boil | 2.7 (16) | 2.8 (5) | 2.9 (11) | 0.93 |
| Filter | 0.2 (1) | 0.6 (1) | 0 | 0.30 |
| Other | 0.2 (1) | 0 | 0.2 (1) | 1.0 |
| Don’t know / no reply | 18.9 (111) | 9.6 (17) | 22.9 (94) | <0.001 |
| **Purpose of water, % (n)** |  |  |  |  |
| Drinking | 43.0 (253) | 57.3 (102) | 36.7 (151) | **<0.001** |
| Handwashing | 78.8 (464) | 84.8 (151) | 76.2 (313) | **0.02** |
| Cleaning | 55.2 (325) | 70.8 (126) | 48.4 (199) | **<0.001** |
| Don’t know / no reply | 17.8 (105) | 9.6 (17) | 21.4 (88) | 0.001 |
| **Sanitation indicators** | | | | |
| **School free of faeces, %(n)** |  |  |  | 0.62 |
| Yes | 81.0 (477) | 83.2 (148) | 80.1 (329) |  |
| No | 15.1 (89) | 12.9 (23) | 16.1 (66) |  |
| No reply / not observed | 3.9 (23) | 3.9 (7) | 3.9 (16) |  |
| **School free of urine, %(n)** |  |  |  | 0.47 |
| Yes | 76.9 (453) | 79.2 (141) | 75.9 (312) |  |
| No | 18.2 (107) | 17.4 (31) | 18.5 (76) |  |
| No reply / not observed | 4.9 (29) | 3.4 (6) | 5.6 (23) |  |
| **Toilets available, %(n)** |  |  |  | **<0.001** |
| Yes | 60.3 (355) | 78.1 (139) | 52.6 (216) |  |
| No | 38.0 (224) | 21.4 (38) | 45.3 (186) |  |
| Don’t know / no reply | 1.7 (10) | 0.6 (1) | 2.2 (9) |  |
| **Toilets functional, %(n)** | N=355 | N=139 | N=216 | 0.41 |
| All | 75.2 (267) | 77.0 (107) | 74.1 (160) |  |
| Some | 14.7 (52) | 15.8 (22) | 13.9 (30) |  |
| None | 3.9 (14) | 3.6 (5) | 4.2 (9) |  |
| No reply / not observed | 6.2 (22) | 3.6 (5) | 7.9 (17) |  |
| **Why toilets not available, %(n)** | N=224 | N=38 | N=186 |  |
| Don’t have toilet facilities | 66.1 (148) | 63.2 (24) | 66.7 (124) | 0.68 |
| No water | 13.0 (29) | 7.9 (3) | 14.0 (26) | 0.43 |
| Toilet is dirty | 4.5 (10) | 5.3 (2) | 4.3 (8) | 0.68 |
| Toilet is broken | 6.7 (15) | 7.9 (3) | 6.5 (12) | 0.72 |
| Toilet is full | 2.2 (5) | 5.3 (2) | 1.6 (3) | 0.20 |
| Toilet only for teachers | 4.5 (10) | 5.3 (2) | 4.3 (8) | 0.68 |
| Other | 5.8 (13) | 7.9 (3) | 5.4 (10) | 0.47 |
| Don’t know / no reply | 6.7 (15) | 5.3 (2) | 7.0 (13) | 1.0 |
| **Year toilets built, %(n)** | N=355 | N=139 | N=216 | 0.09 |
| <2014 | 43.9 (156) | 41.0 (57) | 45.8 (99) |  |
| ≥2014 | 50.4 (179) | 56.1 (78) | 46.8 (101) |  |
| No reply | 5.6 (20) | 2.9 (4) | 7.4 (16) |  |
| **Type of toilet, %(n)** | N=355 | N=139 | N=216 |  |
| Flush toilet | 8.5 (30) | 7.9 (11) | 8.8 (19) | 0.77 |
| Pit latrine | 64.5 (229) | 69.1 (96) | 61.6 (133) | 0.15 |
| Composting toilet | 10.4 (37) | 11.5 (16) | 9.7 (21) | 0.59 |
| Bucket latrine | 3.7 (13) | 0.7 (1) | 5.6 (12) | **0.02** |
| Hanging latrine | 0.3 (1) | 0 | 0.5 (1) | 1.0 |
| No structure | 0.9 (3) | 0 | 1.4 (3) | 0.28 |
| Other | 9.3 (33) | 7.9 (11) | 10.2 (22) | 0.47 |
| Don’t know / No reply | 2.5 (9) | 2.2 (3) | 2.8 (6) | 1.0 |
| **Door on toilet, %(n)** | N=355 | N=139 | N=216 | 0.17 |
| All | 33.5 (119) | 40.3 (56) | 29.2 (63) |  |
| Some | 7.0 (25) | 5.8 (8) | 7.9 (17) |  |
| None | 15.5 (55) | 13.0 (18) | 17.1 (37) |  |
| Don’t know / No reply | 43.9 (156) | 41.0 (57) | 45.8 (99) |  |
| **Door able to lock, %(n)** | N=355 | N=139 | N=216 | 0.09 |
| All | 48.7 (173) | 54.7 (76) | 44.9 (97) |  |
| Some | 13.8 (49) | 11.5 (16) | 15.3 (33) |  |
| None | 35.8 (127) | 33.8 (47) | 37.0 (80) |  |
| Don’t know / No reply | 1.7 (6) | 0 | 2.8 (6) |  |
| **Hygiene indicators** | | | | |
| **Handwashing facilities available, %(n)** |  |  |  | **0.003** |
| Yes | 90.3 (532) | 96.6 (172) | 87.6 (360) |  |
| No | 8.0 (47) | 2.8 (5) | 10.2 (42) |  |
| Don’t know / No reply | 1.7 (10) | 0.6 (1) | 2.2 (9) |  |
| **Type of handwashing facility, %(n)** | N=532 | N=172 | N=360 |  |
| Sink with tap | 1.3 (7) | 1.2 (2) | 1.4 (5) | 0.83 |
| Water tank with tap | 2.8 (15) | 4.7 (8) | 1.9 (7) | 0.08 |
| Tippy tap | 22.0 (117) | 39.5 (68) | 13.6 (49) | **<0.001** |
| Bucket/container with tap | 86.7 (461) | 86.1 (148) | 86.9 (313) | 0.78 |
| Bucket/container without tap | 11.1 (59) | 9.3 (16) | 11.9 (43) | 0.36 |
| Other | 0.9 (5) | 0 | 1.4 (5) | 0.18 |
| Don’t know / No reply | 0 | 0 | 0 | 0 |
| **Handwashing elements, %(n)** | N=532 | N=172 | N=360 |  |
| Soap | 56.8 (302) | 58.1 (100) | 56.1 (202) | 0.66 |
| Soapy water | 66.2 (352) | 64.5 (111) | 66.9 (241) | 0.58 |
| Ash | 0.6 (3) | 0.6 (1) | 0.6 (2) | 1.0 |
| Other | 1.3 (7) | 1.7 (3) | 1.1 (4) | 0.69 |
| Don’t know / No reply | 1.1 (6) | 0 | 1.7 (6) | 0.18 |
| **Hygiene club available, %(n)** |  |  |  | **<0.001** |
| Yes | 54.8 (323) | 66.9 (119) | 49.6 (204) |  |
| No | 43.5 (256) | 32.6 (58) | 48.2 (198) |  |
| Don’t know / no reply | 1.7 (10) | 0.6 (1) | 2.2 (9) |  |

More than one response is possible for some items. ^a^Chi-square or Fisher’s exact test performed to compare responses between WASH supported and WASH unsupported groups. WASH = water, sanitation and hygiene.
